# Wedge‐Like Microstructure of Al_2_O_3_/i‐Ti_3_C_2_T_x_ Electrode with “Nano‐Pumping” Effect for Boosting Ion Diffusion and Electrochemical Defluoridation

**DOI:** 10.1002/advs.202411659

**Published:** 2024-11-22

**Authors:** Junce Wang, Jinfeng Chen, Ningning Liu, Jingjing Lei, Hong‐Wen Gao, Fei Yu, Fanghui Pan, Jie Ma

**Affiliations:** ^1^ Water Resources and Water Environment Engineering Technology Center, Xinjiang Key Laboratory of Engineering Materials and Structural Safety, School of Civil Engineering Kashi University Kashi 844000 P. R. China; ^2^ Research Center for Environmental Functional Materials State Key Laboratory of Pollution Control and Resource Reuse College of Environmental Science and Engineering Tongji University Shanghai 200092 P. R. China; ^3^ College of Oceanography and Ecological Science Shanghai Ocean University No 999, Huchenghuan Road Shanghai 201306 P. R. China

**Keywords:** capacitive deionization, fluorine removal, incomplete etching, intercalation pseudocapacitance, MXene

## Abstract

Controlled synthesis and regulation of 2D nanomaterials with sufficient active sites are promising in electrochemical fluorine capture, but simultaneously achieving rapid rates and efficient activity of intercalation materials remains challengs. Herein, an integrated strategy of micro‐regulation interlayer space and in situ modification of MXenes is proposed to enhance ion storage kinetics. The wedge‐like microstructure of aluminum oxide/incomplete‐Ti_3_C_2_T_x_ MXene (Al_2_O_3_/i‐Ti_3_C_2_ T_x_) is constructed by incomplete etching MAX and in situ derivation of A‐layer element, in which the sub‐nanoscale interlayer space is conducive to the small size ions intercalation, and the formation of “nanopump‐like” effect boosted the ions diffusion. As evidenced by simulation calculations, Al_2_O_3_ nanoparticles not only shorten the migration distance of electrons/hydrated ions in interlayers but also contribute a lower adsorption energy barrier, bringing excellent capture kinetics and stability. Benefiting from the interfacial conversion‐intercalation pseudocapacitance, such electrode is endowed with a high defluoridation capacity (69.9 mg g^−1^ at 1.6V) and an outstanding instantaneous adsorption rate (9.51 mg g^−1^ min^−1^), and shows satisfactory stability in more than 200 cycles. The physicochemical coupling strategy opens a novel approach to optimizing the interlayer structure and in situ modification interface of MXene, which also provids a universal idea for efficient capture of varisized ions of intercalation materials.

## Graphical abstract

Precise regulation of the interlayer space at the nanoscale of Ti_3_C_2_T_x_ MXene according to the size of target ions helps to provide specialized accessibility sites and channels for fluorine capture. The structure optimization of Al_2_O_3_/i‐Ti_3_C_2_T_x_ and Ti_3_C_2_T_x_ and the comparison of F^−^ adsorption energy between them, the “nanopump‐like” effect of the wedge‐like microstructure accelerates the rapid F^−^ diffusion.

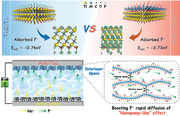

## 1 Introduction

Serious groundwater fluorine pollution has become a threat to the safety of drinking water for the ever‐growing human society.^[^ ^1^ ^]^ Notably, fluorine exists in the form of ions or combined with protons in acidic to neutral environments, while complexes are formed in strongly alkaline water media, and the smaller radius relative to water molecules makes it hard to be target captured, which poses a challenge to separation.^[^ ^2^ ^]^ Current widely applied deionization technologies (including adsorption, precipitation, reverse osmosis membrane, etc.) exist the problems of high pH dependency needs, plus a large number of chemical reagents, low selectivity, and fluorine ions escape, limiting their development in defluoridation.^[^ ^3^ ^]^ Therefore, the design of more economically efficient fluorine capture technology with a low environmental footprint is urgently needed.

Electrochemical fluorine capture (EFC) technology has emerged as a competitive fluorine ions (F^−^) capture technology due to its low energy consumption, excellent regeneration, high removal capacity, and user‐friendly control.^[^ ^4^ ^]^ Based on different fluorine capture mechanisms, electrode materials can be categorized into electrical double layers (EDLs) and pseudocapacitors. In EDLs systems, fluorine storge via fast migration kinetics but suffers from insufficient removal capacity (hardly higher than 20 mg_F_^−^ g^−1^) caused by strong co‐ion repulsion and limited active sites.^[^ ^5^ ^]^ Battery electrodes based on redox reactions can reach an ultra‐high capacity, but the drawbacks of slow reaction kinetics and unsatisfactory reversibility hinder their industrial application.^[^ ^6^ ^]^ In contrast, intercalation pseudocapacitive electrodes promote reversible ion embedding and ejection through lamellar space, which is theoretically superior in terms of ion dynamics and cycling stability.^[^ ^7^ ^]^ 2D layered materials are commonly utilized as intercalation pseudocapacitors to capture ions, such as layered double hydroxides and oxides (LDHs/LDOs), transition metal dichalcogenides (TMDs), and MXenes. Among them, LDHs/LDOs have been extensively researched but are still restricted by slow mass transfer and reaction kinetics.^[^ ^8^ ^]^ TMDs will inevitably cause severe capacity attenuation caused by structural deterioration in the irreversible phase transitions.^[^ ^9^ ^]^ In this case, how to design the high‐capacitance and fast‐rate intercalation materials is still a challenge and has rarely been reported in electrochemical defluoridation so far.

Transition metal carbides and nitrides (MXenes) offer a more attractive platform over other 2D materials for ion capture and storage applications.^[^ ^10^ ^]^ The M_n+1_X_n_T_x_ layers are weakly bonded by the van der Waals force, resulting in MXenes with a more controllable intercalation structure to increase capacity or alleviate expansion and stacking.^[^ ^11^ ^]^ Benefiting from the inherent transition metal chemistry and different conductive network structures, MXenes can adjust their electronic properties from metals to semiconductors (electrical conductivity up to 10 000 S cm^−1^).^[^ ^12^ ^]^ Finally, the rich surface functional groups endow MXenes with ideal hydrophilicity and more diverse modification possibilities, making them host materials for electrochemical ion capture/deintercalation.^[^ ^13^ ^]^ Typically, common reports have been based on expanding the layer spacing strategy to create more effective capture sites and improve the storage performance of large‐sized ions (such as Na^+^ and Cl^−^).^[^ ^13a^ ^]^ Nevertheless, the smaller ions (such as F^−^) are much less competitive for the active site than the larger ones in that case. Precise regulation of the interlayer space at the nanoscale according to the size of target ions helps to provide specialized accessibility sites and channels for targeted capture.^[^ ^14^ ^]^ In addition, A‐layer elements with diverse chemical identities can in situ modify the MXenes to improve their electrochemical activity and interlayer spaces in a the form of oxides, which has been almost ignored in previous studies.^[^ ^15^ ^]^ For example, Zhang et al., demonstrated that Al_2_O_3_ had a strong affinity for electrochemical F^−^ capture, which inspired the efficient use of Al element in Ti_3_AlC_2_ to design MXene‐based defluoridation electrode materials.^[^ ^16^ ^]^ Meanwhile, the construction of MXene composites on the microscopic scale is helpful to strengthen the host structure and improve the cycle stability.^[^ ^17^ ^]^ It is of great research value to combine reasonable regulation of interlayer space and effective utilization of A‐layer elements to maximize the release of the electrochemical storage potential of MXene and realize the rapid capture of target ions.

Herein, we proposed an integrated strategy to construct the wedge‐like microstructure of aluminum oxide/incomplete‐Ti_3_C_2_T_x_ MXene (Al_2_O_3_/i‐Ti_3_C_2_T_x_) to achieve tiny regulation of the interlayer space (from 9.17 to 11.81 Å) by incompletely etching and in situ derivation. Based on the different widths and narrowness of its interlayer, the formation of a “nanopump‐like” effect boosted the rapid transport and capture of ions and electrons. The derivation of Al_2_O_3_ provided additional Al‐F selective capture sites, allowing Al_2_O_3_/i‐Ti_3_C_2_T_x_ to exhibit excellent defluoridation properties. The interfacial conversion‐intercalation pseudocapacitive capture mechanism of fluorine was revealed. Moreover, Density Functional Theory (DFT) and Staircase Potential Electrochemical Impedance Spectroscopy (SPEIS) further confirmed its enhanced F^−^ storage kinetics via ultralow adsorption energy between Al─O─Al and F^−^. Based on the above advantages, the EFC system with high fluorine capture capacity (69.9 mg g^−1^ at 1.6V), rapid instantaneous removal rate (9.51 mg g^−1^ min^−1^), and durability can be realized by taking Al_2_O_3_/i‐Ti_3_C_2_T_x_ as anode material. Our strategy of incomplete etching combined recycle of Al‐elements provides a feasible scheme for micro‐regulating interlayer space and modifying MXene, which is expected to be extended to the electrode design of electrochemical capture for ions in different sizes.

## 2 Results and Discussion

### 2.1 Preparation and Characterization of Al_2_O_3_/i‐Ti_3_C_2_T_x_

**Figure 1:**
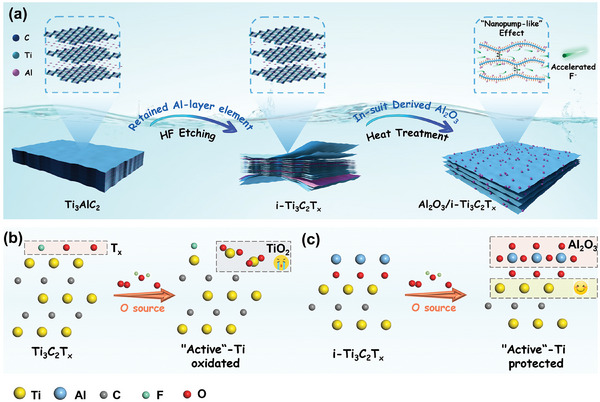
a) Schematic diagram of the synthesis process of Al_2_O_3_/i‐Ti_3_C_2_T_x_ composites. The theoretical evolution of b) Ti_3_C_2_T_x_ and c) i‐Ti_3_C_2_T_x_ during oxidation.

**Figure** **1**a illustrates the facile synthesis procedure of Al_2_O_3_/i‐Ti_3_C_2_T_x_. In brief, Al_2_O_3_/i‐Ti_3_C_2_T_x_ was synthesized through annealing treatment on the basis of incomplete HF‐etching Ti_3_AlC_2_. Further, i‐Ti_3_C_2_T_x_ with different etching degrees can be obtained by adjusting the concentration of HF. The Ti with different surface functional groups in Ti_3_C_2_T_x_ can be divided into an inert state (C─Ti─F) and an active state (C─Ti─O). As shown in Figure 1b, the active‐Ti was often more easily oxidized to TiO_2_ when “attacked” by the oxygen atmosphere. In this work, the aluminum sources of Al_2_O_3_ derived from i‐Ti_3_C_2_T_x_ included the residual Al‐atoms between Ti‐C layers and the free Al‐atoms adsorbed by surface ‐O. According to oxygen diffusion theory,^[^ ^18^ ^]^ the surface free Al‐element with lower standard redox potential (−1.66V, while Ti^2+^/Ti is −0.36V) preferentially combined with oxygen (provided by interlayer water from i‐ Ti_3_C_2_T_x_) to derive Al_2_O_3_, which effectively protected the inner Ti‐layer (Figure 1c).

**Figure 2:**
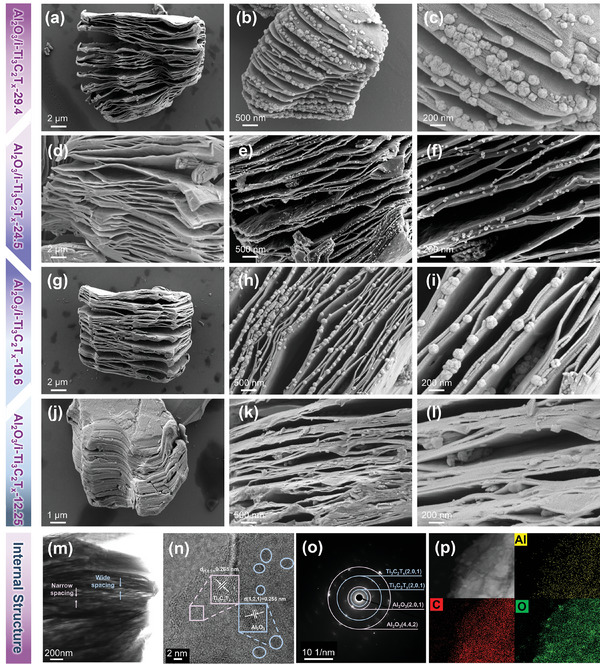
Morphology and structural characterizations. SEM images of a) i‐Ti_3_C_2_T_x_‐29.4, b,c) Al_2_O_3_/i‐Ti_3_C_2_T_x_‐29.4, d) i‐Ti_3_C_2_T_x_‐24.5, e,f) Al_2_O_3_/i‐Ti_3_C_2_T_x_‐24.5, g) i‐Ti_3_C_2_T_x_‐19.6, h,i) Al_2_O_3_/i‐Ti_3_C_2_T_x_‐19.6, j) i‐Ti_3_C_2_T_x_‐12.25, k,l) Al_2_O_3_/i‐Ti_3_C_2_T_x_‐12.25. m) HAADF‐STEM, n) HRTEM, o) SAED pattern, p) HAADF image and elemental mapping images of Al, C, and O elements for Al_2_O_3_/i‐Ti_3_C_2_T_x_‐24.5.

Scanning electron microscopy (SEM) and transmission electron microscopy (TEM) were utilized to reveal the microscopic morphology and internal structure of as‐prepared materials. The etching degree of Ti_3_AlC_2_ deepened with the increase of HF concentration and dosage and evolved from stepped block to complete accordion‐like (**Figure** **2a,d,g,j**; Figure S1a,c, Supporting Information). In Figure 2e,f, the Al_2_O_3_/i‐Ti_3_C_2_T_x_‐24.5 (representing HF concentration of 24.5%) featured twisted wedge‐like architecture loading with uniform nanoparticles (NPs) after calcination. The NPs size investigation for Al_2_O_3_/i‐Ti_3_C_2_T_x_‐24.5 displayed in Figure S2 (Supporting Information) revealed that their diameter distribution ranged from 40 to 50 nm. The SEM images of Al_2_O_3_/i‐Ti_3_C_2_T_x_‐29.4 (Figure 2b,c) and Al_2_O_3_/i‐Ti_3_C_2_T_x_‐19.6 (Figure 2h,i) showed that larger NPs (100–200 nm) were dispersed on i‐Ti_3_C_2_T_x_ substrate, and the former appeared to be significantly agglomerated. This phenomenon was due to excessive etching resulting in more aluminum sources on the surface, thus sufficient nucleation to grow agglomerated large‐size NPs. Notably, no obvious NPs were derived when the degree of etching was too low or high (Al_2_O_3_/i‐Ti_3_C_2_T_x_‐12.25 in Figure 2k,i, i‐Ti_3_C_2_T_x_‐36.75 in Figure S1b, Ti_3_C_2_T_x_ in Figure S1d, Supporting Information), which can be attributed to the lack of free‐aluminum content residue. Besides, high‐angular annular dark‐field scanning TEM (HAADF‐STEM) demonstrated Al_2_O_3_/i‐Ti_3_C_2_T_x_‐24.5 showed a wedge‐like microstructure with interlayer spacing varying in width (Figure 2m). As shown in Figure 2n, two different classes of labeled regions and profiles showed lattice fringes with 0.255 and 0.265 nm, corresponding to the (121) plane of Al_2_O_3_ and the (101) plane of Ti_3_C_2_T_x_. The selected area electron diffraction (SAED) pattern presented distinct diffraction rings related to (101), (201) of Ti_3_C_2_, and (201), (442) of Al_2_O_3_ (Figure 2o). HAADF and elemental mappings images shown in Figure 2p and Figure S3 (Supporting Information) displayed the homogenous distribution of Al and O on the Ti, C in i‐Ti_3_C_2_T_x_. Based on the above analysis, the i‐Ti_3_C_2_T_x_ with different free‐aluminum sources were controllably synthesized under changing proportioned etching conditions, and Al_2_O_3_ NPs of various sizes and contents can be derived after calcination. Importantly, the introduction of Al_2_O_3_ into the MXene matrix can produce more electrochemically active sites, and the wedge‐shaped Al_2_O_3_/i‐Ti_3_C_2_T_x_‐24.5 produced a “nanopump‐like” effect to enhance the ion storage dynamics.

**Figure 3:**
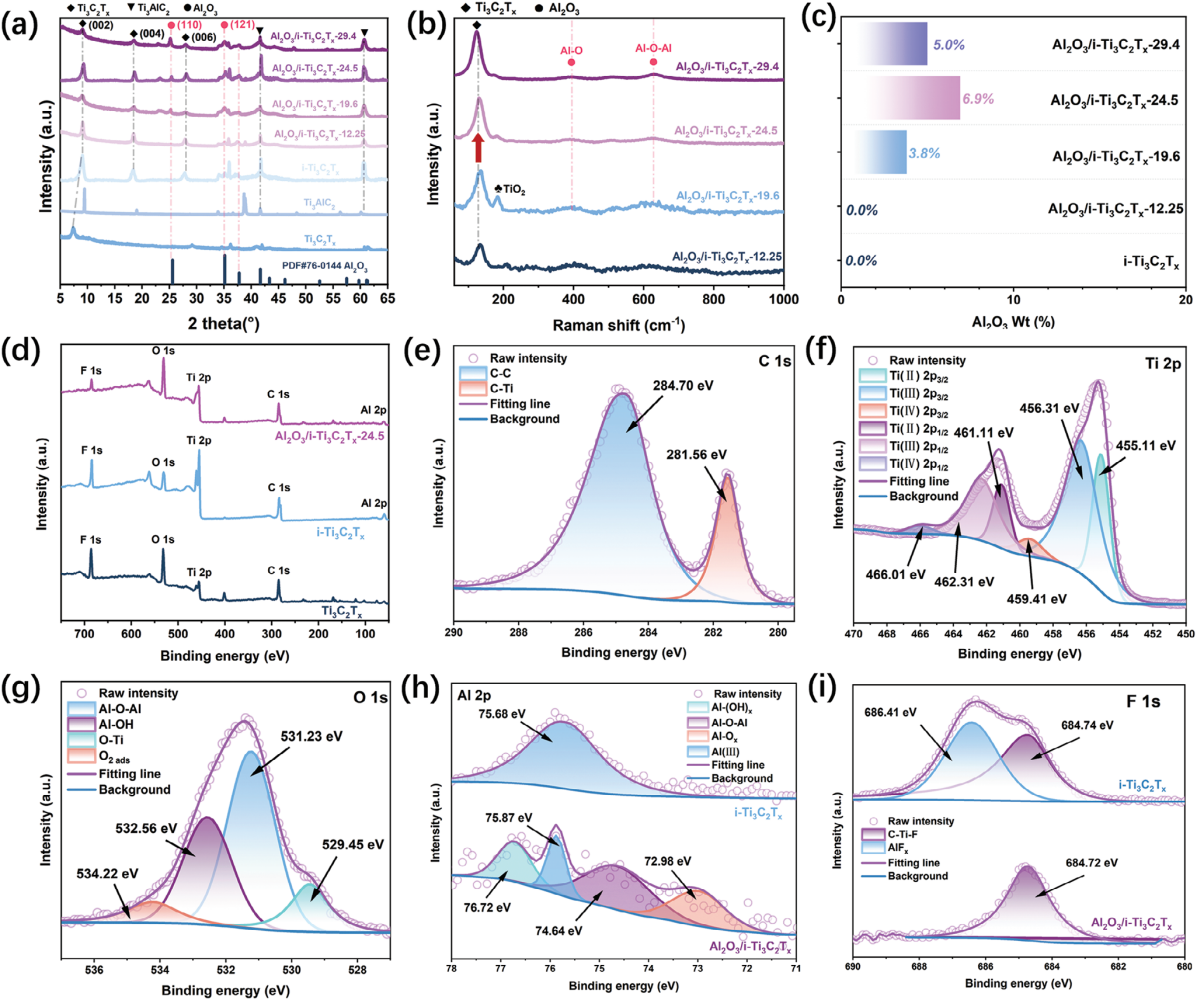
a) XRD patterns of Ti_3_AlC_2_, Ti_3_C_2_T_x_, i‐Ti_3_C_2_T_x_, and Al_2_O_3_/i‐Ti_3_C_2_T_x_‐29.4/24.5/19.6/12.25. b) Raman spectra of Al_2_O_3_/i‐Ti_3_C_2_T_x_‐29.4/24.5/19.6/12.25. c) Al_2_O_3_ wt. of i‐Ti_3_C_2_T_x_ and Al_2_O_3_/i‐Ti_3_C_2_T_x_‐29.4/24.5/19.6/12.25. d) XPS survey spectrum of Ti_3_C_2_T_x_, i‐Ti_3_C_2_T_x_, and Al_2_O_3_/i‐Ti_3_C_2_T_x_‐24.5. XPS spectra of e) C 1s, f) Ti 2p, g) O 1s of Al_2_O_3_/i‐Ti_3_C_2_T_x_‐24.5 and h) Al 2p, i) F 1s of i‐Ti_3_C_2_T_x_ and Al_2_O_3_/i‐Ti_3_C_2_T_x_‐24.5.

The crystal phase and structure characteristics of the prepared i‐Ti_3_C_2_T_x_ and their derivatives were determined by XRD. As shown in **Figure** **3**a, i‐Ti_3_C_2_T_x_ had prominent peaks with all Al_2_O_3_/i‐Ti_3_C_2_T_x_ samples at 9.1°, 18.5°, and 28°, which can be well indexed with the (002), (004), and (006) crystal planes of the Ti_3_C_2_T_x_ structure. Meanwhile, their peaks at 41.6° and 60.6° matched the characteristic peaks of Ti_3_AlC_2_. This indicates that the i‐Ti_3_C_2_T_x_ obtained by incomplete etching was between multilayer MXene and massive MAX.^[^ ^19^ ^]^ More importantly, the new peaks at 25.6°, 35.1°, 37.8° of Al_2_O_3_/i‐Ti_3_C_2_T_x_‐29.4/24.5/19.6 were highly consistent with the (110), (121) and (‐110) planes of Al_2_O_3_ (PDF#76‐1044). Al_2_O_3_/i‐Ti_3_C_2_T_x_‐12.25 showed no corresponding characteristic peaks of Al_2_O_3_, which was consistent with the result that no NPs appeared in the above SEM images. Therefore, it can be determined the phase of the as‐fabricated products mainly consisted of Ti_3_C_2_T_x_ substrate and Al_2_O_3_ NPs. In Raman spectra (Figure 3b), Al_2_O_3_/i‐Ti_3_C_2_T_x_‐29.4/24.5/19.6 showed the stretching vibration at 398 cm^−1^ of asymmetric Al─O and 622 cm^−1^ of O─Al─O, while no obvious Al_2_O_3_ Raman peaks appeared in Al_2_O_3_/i‐Ti_3_C_2_T_x_‐12.25. With the increase of HF concentration, the format of all samples with respect to Ti_3_C_2_T_x_ few layers were gradually enhanced, which indicated the incomplete etching degree of Ti_3_C_2_T_x_ had been successfully regulated. The Al_2_O_3_ content in all samples was determined by ZnO internal standard analysis, which was 5.0% of Al_2_O_3_/i‐Ti_3_C_2_T_x_‐29.4, 6.9% of Al_2_O_3_/i‐Ti_3_C_2_T_x_‐24.5 and 3.8% of Al_2_O_3_/i‐Ti_3_C_2_T_x_‐19.6 (Figure 3b; Figure S4, Supporting Information). The weight contents (wt. %) of the Al element in the samples were further determined by ICP‐OES, and the overall results were similar to those of the XRD internal standard (Table S1, Supporting Information). It is worth noting that although Al_2_O_3_/i‐Ti_3_C_2_T_x_‐12.25 has a high Al‐element weight content in ICP‐OES results, the characteristic peak of Al_2_O_3_ cannot be identified in the XRD results. This can be explained by the previous oxygen diffusion oxidation theory: i‐Ti_3_C_2_T_x_‐12.25 with too low etching degree still maintained a high MAX structure, and Al‐element was enclosed between the Ti layer and the C layer so that no free‐aluminum source was derived on the surface.

XPS spectra (Figure 3d–i; Figure S5, Supporting Information) were adopted to analyze the surface composition and elemental oxidation state of i‐Ti_3_C_2_T_x_ and Al_2_O_3_/i‐Ti_3_C_2_T_x_. Figure 3d explained the coexistence of F 1s, O 1s, Ti 2p, and C 1s in Ti3C2Tx, while i‐Ti_3_C_2_T_x_ and Al_2_O_3_/i‐Ti_3_C_2_T_x_‐24.5 additionally recognized Al 2p. Notably, the F 1s peak strength of i‐Ti_3_C_2_T_x_ decreased significantly after calcination, where the less of ‐F terminal helped to improve the hydrophilicity and charge transfer ability of Al_2_O_3_/i‐Ti_3_C_2_T_x_‐24.5.^[^ ^16^ ^]^ In Figure 3e and Figure S5a (Supporting Information), C─C (284.70 eV) and C─Ti (281.56 eV) bonds proved the samples retained the complete structure of Ti_3_C_2_T_x_.^[^ ^20^ ^]^ Peaks at 455.11, 456.31, and 461.11 eV were indexed as Ti (II), Ti (III), and Ti (IV) (Figure 3f), corresponding to carbon‐bonded titanium atoms with terminals and oxidized titanium (C─Ti─F, C─Ti─O, and O─Ti─O).^[^ ^21^ ^]^ Four main peaks at 529.45, 531.23, 532.56, and 534.22 eV in the O 1s spectrum (Figure 3g) were related to functionalized C‐Ti‐O_x_, Al─O─Al, Al‐(OH)_x_, and absorbed O_2_, respectively.^[^ ^22^ ^]^ In the XPS spectra of Al 2p (Figure 3h), Al(III) (75.67 eV) was shown in i‐Ti_3_C_2_T_x_, while Al_2_O_3_/i‐Ti_3_C_2_T_x_‐24.5 additions of 72.98, 74.64, and 76.72 eV can be attributed to Al─O_x_, Al─O─Al, and Al‐(OH)_x_.^[^ ^23^ ^]^ The strong component at 684.72 eV of i‐Ti_3_C_2_T_x_ and Al_2_O_3_/i‐Ti_3_C_2_T_x_‐24.5 belonged to the C─Ti─F_x_ bonds and a weak shoulder fingerprint of AlF_x_ at 686.41 eV of i‐Ti_3_C_2_T_x_ (Figure 3i).^[^ ^21^ ^]^ The above characterization confirmed the trance existence of the Al_2_O_3_ phase and the structural integrity of i‐Ti_3_C_2_T_x_ before and after calcination.

### 2.2 Electrochemical Performance and Ion Storage Kinetic of Al_2_O_3_/i‐Ti_3_C_2_T_x_

**Figure 4:**
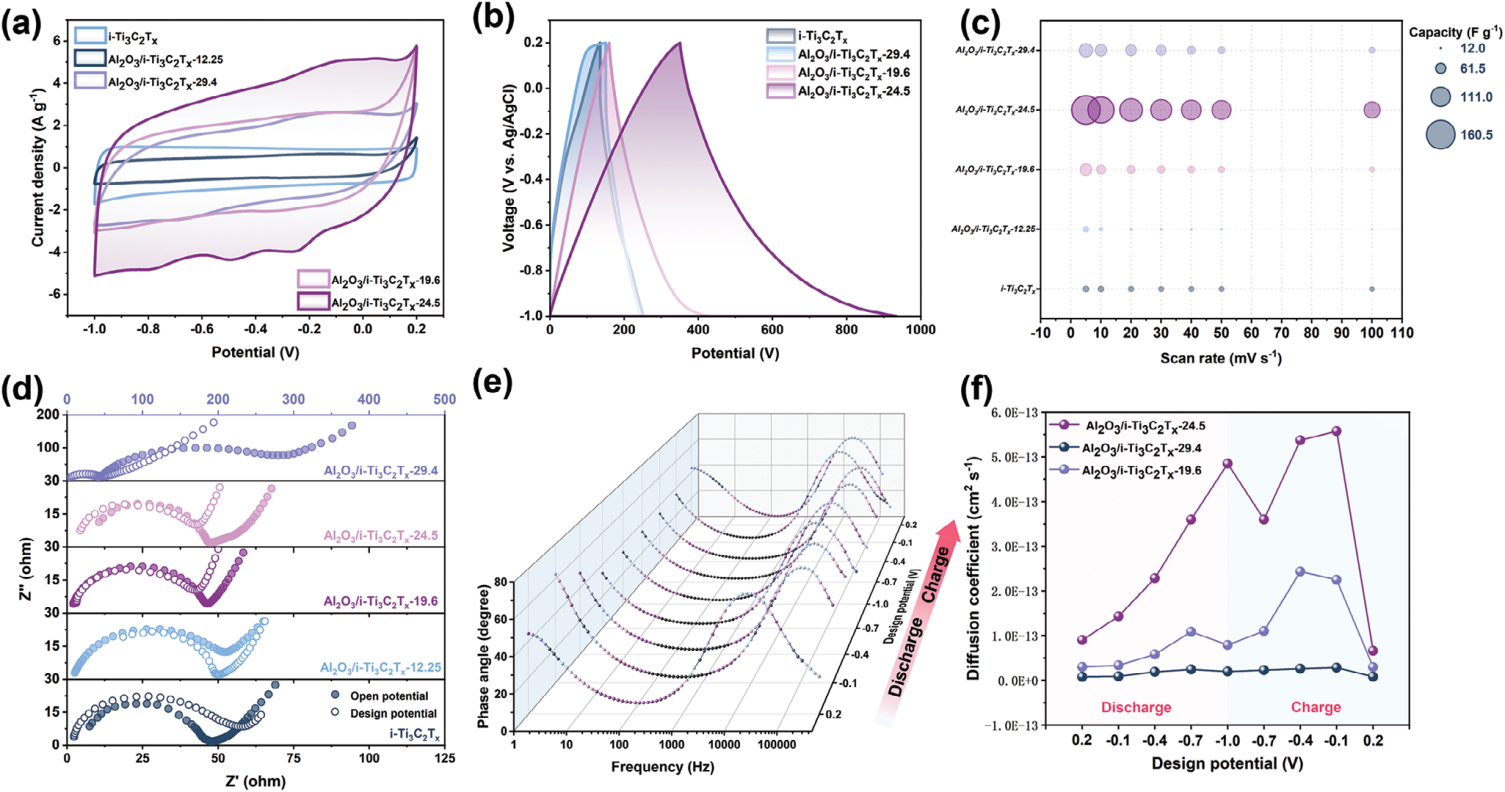
a) CV curves of Al_2_O_3_/i‐Ti_3_C_2_T_x_‐29.4/24.5/19.6/12.25 and i‐Ti_3_C_2_T_x_ at a scan rate of 30 mV s^−1^, b) GCD curves of the as‐prepared samples at current density of 100 mA g^−1^, c) Specific capacitance of Al_2_O_3_/i‐Ti_3_C_2_T_x_‐29.4/24.5/19.6/12.25 and i‐Ti_3_C_2_T_x_ at different scan rates, d) Nyquist plots of the as‐prepared samples at open/design potentials, e) Bode plots for Al_2_O_3_/i‐Ti_3_C_2_T_x_‐24.5 during charge–discharge between −0.8 and 0.2 V, f) diffusion coefficient at −0.8–0.2 V during charge–discharge cycle.

The electrochemical properties of the above five products were identified by cycle voltammetry (CV), and the curves at different scan rates are shown in Figure S6 (Supporting Information). **Figure** **4**a compared the CV curves of different samples at a scan rate of 30 mV s^−1^, Al_2_O_3_/i‐Ti_3_C_2_T_x_‐29.4/24.5/19.6 displayed the distorted rectangle‐like curve and no redox peaks appeared, which indicated the ion storage mechanism led by pseudocapacitance. A slight fluctuation in the CV curve of Al_2_O_3_/i‐Ti_3_C_2_T_x_‐24.5 was observed, which may be caused by the violent ion intercalation process. Correspondingly, the GCD curves of Al_2_O_3_/i‐Ti_3_C_2_T_x_‐24.5 without platform at different current densities reflected that the conversion capture of F^−^ occurred at the interface rather than inside (Figure S7, Supporting Information). Lower current density would prolong the charge/discharge time, that is, more active sites were activated to capture ions,^[^ ^24^ ^]^ thus the longest charge/discharge time of Al_2_O_3_/i‐Ti_3_C_2_T_x_‐24.5 confirmed its amazing electrochemical reaction capacity (Figure 4b). By integrating the CV closed curve, Al_2_O_3_/i‐Ti_3_C_2_T_x_‐24.5 obtained a high specific capacitance of 160.2 F g^−1^, 3–5 times higher than other samples (following the sequence of Al_2_O_3_/i‐Ti_3_C_2_T_x_‐24.5 > Al_2_O_3_/i‐Ti_3_C_2_T_x_‐29.4 > Al_2_O_3_/i‐Ti_3_C_2_T_x_‐19.6 ⪢ i‐Ti_3_C_2_T_x_ > Al_2_O_3_/i‐Ti_3_C_2_T_x_‐12.25) (Figure 4c). The pseudocapacitance or diffusion properties of Al_2_O_3_/Ti_3_C_2_ can be further explored through experimental data analysis (Figure S8, Supporting Information), which showed that Al_2_O_3_/i‐Ti_3_C_2_T_x_‐24.5 owned strong pseudocapacitance dominant behavior. The above analysis is sufficient to demonstrate that Al_2_O_3_/i‐Ti_3_C_2_T_x_‐24.5 coupled MXene‐intercalation and Al_2_O_3_ NPs‐interfacial transformation mechanism, thus showing great electrochemical reaction potential.

In order to fully analyze the kinetic information of the samples and the relationship between it and electrochemical defluoridation, the dynamic impedance within the voltage window (−0.8–0.2 V) cycle and under the operating voltage (1.4 V) was studied by SPEIS.^[^ ^25^ ^]^ Figure 4d compared the Nyquist diagram of each sample at open/design potential, respectively. When charged to 1.4 V, the semicircle radius in the high‐frequency region became smaller and the linear slope in the low‐frequency region became steeper, which indicates that the interface charge transfer resistance decreases rapidly under the design voltage (Figure S9, Supporting Information). As shown in Table S2 (Supporting Information), had the lowest R_ct_ (30.46 Ω), demonstrating its excellent charge transfer capability under operating conditions. Figure S10 (Supporting Information) gave the Nyquist plots at staircase potentials of Al_2_O_3_/i‐Ti_3_C_2_T_x_‐24.5/29.4/12.25 during the charge–discharge cycle. All samples can be observed to consist of concave semicircles and straight lines, indicating the full complexation of the interface and rapid intercalation process. Al_2_O_3_/i‐Ti_3_C_2_T_x_‐29.4 presented two semicircles, which may be caused by the poor contact between the large NPs agglomeration on the surface and the substrate. Besides, the steeper vertical line in the low‐frequency region during charge/discharge indicated that the ion diffusion rate was greatly accelerated, which was the key to ensuring a high rate of fluoride removal. Bode plots (Figure 4e; Figure S11, Supporting Information) further showed such kinetic behavior. Benefiting from the wedge‐like structure, Al_2_O_3_/i‐Ti_3_C_2_T_x_‐24.5/29.4 peaked at high frequencies of ≈100Hz, representing their rapid transfer storage and pseudocapacitance capabilities.^[^ ^25^ ^]^ Importantly, only Al_2_O_3_/i‐Ti_3_C_2_T_x_‐24.5 can still observe ≈45° phase angle at the lowest frequency of 0.1Hz, which indicates that the sample showed desirable rapid ion diffusion.^[^ ^26^ ^]^ According to the calculated Warburg coefficient (Figure 4f; Figure S12, Supporting Information), the diffusion coefficient of the sample at dynamic voltage is semi‐quantitatively estimated. Al_2_O_3_/i‐Ti_3_C_2_T_x_‐24.5 showed the highest ionic diffusion coefficient in the whole process (one order of magnitude higher than Al_2_O_3_/i‐Ti_3_C_2_T_x_‐12.25), which was the reason why Al_2_O_3_/i‐Ti_3_C_2_T_x_‐24.5 was not dominated by diffusion due to its high capacitance ratio. Benefiting from the “nanopump‐like” effect formed by the wedge‐like microstructure, Al_2_O_3_/i‐Ti_3_C_2_T_x_‐24.5 exhibited rapid charge/ion storage kinetics, thereby fully utilizing its electrochemical defluoridation properties.

### 2.3 Defluoridation Performance of Al_2_O_3_/i‐Ti_3_C_2_T_x_

**Figure 5:**
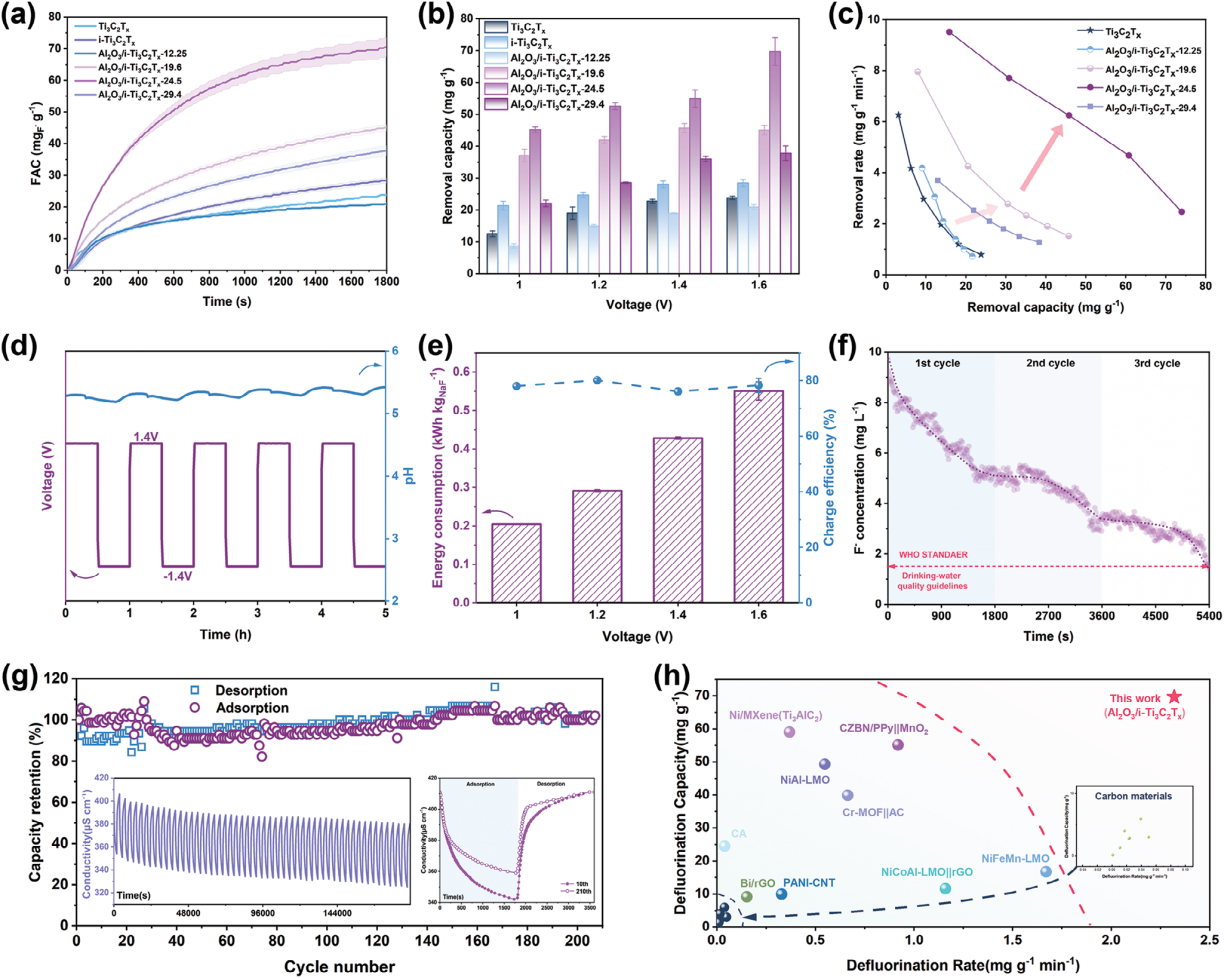
a) FAC vs. time in one cycle (1.4 V, 100 mg L^−1^ F^−^ solution), and b) removal capacity at different operating voltages of Ti_3_C_2_T_x_, i‐Ti_3_C_2_T_x_ and Al_2_O_3_/i‐Ti_3_C_2_T_x_‐29.4/24.5/19.6/12.25. c) Kim–Yoon plots of Ti_3_C_2_T_x_ and Al_2_O_3_/i‐Ti_3_C_2_T_x_‐29.4/24.5/19.6/12.25, d) pH fluctuation during five charge and discharge cycles, e) energy consumption and charge efficiency of Al_2_O_3_/i‐Ti_3_C_2_T_x_‐24.5 at various voltage (1.0–1.4V); f) change of F^−^ concentration during three cycles at a cell voltage of 1.4 V in 10 mg L^−1^ F^−^ solution, g) the long cycle defluoridation performance of Al_2_O_3_/i‐Ti_3_C_2_T_x_‐24.5 at 1.4V, h) The comparison of defluoridation capacity and rate with other reported fluorine removal electrodes.

The fluorine capture process was evaluated in hybrid capacitive deionization (HCDI) (Figures S13 and S14, Supporting Information). In the first 800 s, the fluorine adsorption capacity (FAC) of Al_2_O_3_/i‐Ti_3_C_2_T_x_‐24.5 gradually reached equilibrium with the fastest rising trend, and the order of FAC at 1800 s was as follows: Al_2_O_3_/i‐Ti_3_C_2_T_x_‐24.5 > Al_2_O_3_/i‐Ti_3_C_2_T_x_‐19.6 > Al_2_O_3_/i‐Ti_3_C_2_T_x_‐29.4 > i‐Ti_3_C_2_T_x_ > Ti_3_C_2_T_x_ > Al_2_O_3_/i‐Ti_3_C_2_T_x_‐12.25 (**Figure** **5**a). The FAC of the above samples at different voltages was compared (Figure 5b), and the defluoridation capacity of Al_2_O_3_/i‐Ti_3_C_2_T_x_‐24.5 was the highest at all constant voltages (3‐4 times higher than that of others). According to the maximum allowable charge (MAC) theory,^[^ ^27^ ^]^ high voltage essentially increased the amount of charge transferred to the electrode to enhance its defluoridation ability. At a high constant‐voltage of 1.6 V, the max FAC of Al_2_O_3_/i‐Ti_3_C_2_T_x_‐24.5 reached 69.69 mg g^−1^ in 100 mg L^−1^ NaF feed. Figure 5c depicted the CDI Ragone plots for all samples, and the Al_2_O_3_/i‐Ti_3_C_2_T_x_‐24.5 electrodes simultaneously showed a higher FAC and time‐average removal rate than those of the others. In Figure S15 (Supporting Information), Al_2_O_3_/i‐Ti_3_C_2_T_x_‐24.5 achieved a maximum instantaneous rate (9.51 mg g^−1^ min^−1^) within 200 s, and its average removal rate was 2–3 times faster than others at any voltage, highlighting its excellent electrochemical fluorine capture ability. The stability of the Al_2_O_3_/i‐Ti_3_C_2_T_x_‐24.5 electrode was verified by real‐time monitoring of pH changes in 5 cycles, and the results of slight fluctuations ≈5.3 confirmed the potential of the system for stable defluoridation (Figure 5d). The energy consumption (EC) rose from 0.13 to 0.55 kWh kg_NaF_^−1^ and charge efficiency was range of 67–80% as the voltage interval, which was still a desirable level in EFC (Figure 5e; Figure S16, Supporting Information).

In addition, the applicability of the Al_2_O_3_/i‐Ti_3_C_2_T_x_‐24.5 electrode in simulating complex water conditions was further verified. As shown in Figure S17 (Supporting Information), the Al_2_O_3_/i‐Ti_3_C_2_T_x_‐24.5 electrode maintained ideal removal efficiency when multiple ions coexist. Further, after only 3 cycles at 1.4 V, the F‐ concentration decreased from 10 to 1.364 mg L^−1^, which was lower than the 1.5 mg L^−1^ stipulated in drinking‐water quality guidelines of WHO standard (Figure 5f). The electrochemical fluorine capture/release process was tested for a long run of ≈10 days (Figure 5g). After more than 200 cycles, the capacity retention rate still reached 90%, which indicated that there was no further structural damage in the water. The almost conserved adsorption and desorption capabilities of the system proved that the Al_2_O_3_/i‐Ti_3_C_2_T_x_‐24.5 electrode owned excellent reversible capacity and dynamic equilibrium (Figure 5g, insert). Compared with the previously reported defluorinated electrode materials, the Al_2_O_3_/i‐Ti_3_C_2_T_x_‐24.5 electrode still exhibited exciting performance in terms of FAC and rate (Figure 5h; Table S3, Supporting Information). Therefore, the excellent defluoridation efficiency, long cycle life, and stability in dealing with complex water conditions made it possible for the Al_2_O_3_/i‐Ti_3_C_2_T_x_‐24.5 electrode to expand the purification of low‐concentration fluorinated groundwater.

### 2.4 Mechanistic Analysis of Fluorine Adsorption and Diffusion in Al_2_O_3_/i‐Ti_3_C_2_T_x_

**Figure 6:**
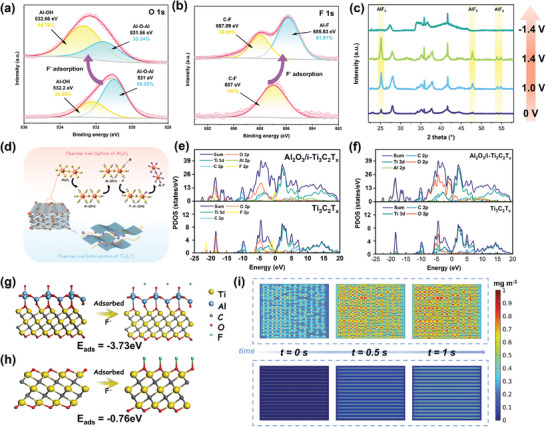
a) O 1s, b) F 1s XPS spectra, and c) heterotopic XRD pattern before and after adsorption of F^−^ in Al_2_O_3_/i‐Ti_3_C_2_T_x_‐24.5 electrode, d) schematic diagram of Al_2_O_3_ and i‐Ti_3_C_2_T_x_ capture F‐ processes, calculated PDOS of Al_2_O_3_/i‐Ti_3_C_2_T_x_ and Ti_3_C_2_T_x_ with Al, Ti, C, O and F orbitals e) after and f) before absorbing F^−^, Side view of the optimized models of g) Al_2_O_3_/i‐Ti_3_C_2_T_x_ and h) Ti_3_C_2_T_x_ before and after absorbing F^−^, i) finite element simulation results for the constant F^−^ concentrations in Al_2_O_3_/i‐Ti_3_C_2_T_x_ and Ti_3_C_2_T_x_ model with time (0–1s).

Exploring the synergistic effect of i‐Ti_3_C_2_T_x_ and Al_2_O_3_ on F^−^ capture was of great significance to fill the gap in the EFC mechanism. Ectopic XPS analysis was used to observe the valence state composition and bonding mode of Al_2_O_3_/i‐Ti_3_C_2_T_x_‐24.5 after F^−^ adsorption/desorption. In **Figure** **6**a, the O 1s spectrum showed that the peak area of Al─O─Al decreased (69.05%–35.24%), while the peak area of Al‐OH increased (30.95%–64.76%), indicating that Al_2_O_3_ was activated to form Al─OH to further complexed with F^−^ through hydrogen bonding.^[^ ^16^ ^]^ The appearance of the Al─F peak after adsorption indicates that a strong bond was formed between fluorine and Al atoms through electron transfer or sharing (Figure 6b).^[^ ^28^ ^]^ After capturing F^−^, the transfer of the O 1 and F 1s bands to higher levels may be caused by electron sharing/transfer between Al/O and F. Further, the phase transition and reversibility of Al_2_O_3_/i‐Ti_3_C_2_T_x_‐24.5 electrode during electrochemical defluoridation were tracked by heterotopic XRD (Figure 6c). At both 1.0 and 1.4 V, two new peaks appeared at 25.3° and 47.9°, indexed to the (100) and (511) planes of the AlF_3_ phase (PDF#83‐0719), respectively. After discharge at −1.4 V, the AlF_3_ peak almost completely disappeared, representing the appreciable reversibility of the Al_2_O_3_/i‐Ti_3_C_2_T_x_‐24.5 electrode. Based on the above analysis, it is inferred that the fluorine capture processes of i‐Ti_3_C_2_T_x_ and Al_2_O_3_ are shown in Figure 6d. Al_2_O_3_ was first activated to AlO (OH), which “grabbed” fluorine through hydrogen bonding, and then Al formed a strong bond with F through electron transfer/sharing to “fix” it. When discharged, the process was reversed and the Al_2_O_3_/i‐Ti_3_C_2_T_x_‐24.5 electrode was recycled. The main process can be shown in the following formula (Equation (1) and (2)):

(1) $$Ti_{3}C_{2}T_{x}+nF^{-}-ne^{-}↔Ti_{3}C_{2}T_{x}F_{n}$$

(2) $$Al_{2}O_{3}\to \mathrm{AlO}(\mathrm{OH})\to \mathrm{AlO}(\mathrm{OH})\cdot \cdot \cdot F\to F-\mathrm{Al}-\left(\mathrm{OH}\right)$$

DFT calculations provided a comprehensive understanding of the atomic‐level mechanism involved in the electrochemical capture and removal of F^−^ from the Al_2_O_3_/i‐Ti_3_C_2_T_x_/F^−^ compound. The enhanced density of states (DOS) of Al_2_O_3_/i‐Ti_3_C_2_T_x_ near the Fermi level indicates that the electronic synergy between Al_2_O_3_ and i‐Ti_3_C_2_T_x_ accelerated charge transfer (Figure S18, Supporting Information).^[^ ^29^ ^]^ In Figure 5e,f, the electron density of Al_2_O_3_/i‐Ti_3_C_2_T_x_ was rearranged after the capture of fluorine. The d orbitals of Ti (Ti 3d), and the p orbitals of O and Al (O 2p and Al 2p) all had overlapping peaks with F 1s, which can be reasonably judged the possibility of bonds forming.^[^ ^30^ ^]^ The highest energy of the bonding orbital at the Al site demonstrated it had a strong adsorption effect on F.^[^ ^31^ ^]^ The optimized Al_2_O_3_/Ti_3_C_2_ and Ti_3_C_2_ models were established in Figure 6g,h. The adsorption energy (E_ads_) of a single F^−^ on i‐Ti_3_C_2_T_x_ and in situ derived Al_2_O_3_ was −0.76 and −3.73 eV, respectively, indicating that Al_2_O_3_ helped to enhance the interface capture of F^−^. The adsorption differences of electron interactions between the two models and F^−^ were further supplemented, where yellow and cyan represent electron accumulation and consumption. Al_2_O_3_/i‐Ti_3_C_2_T_x_ displayed more obvious electron delocalization than Ti_3_C_2_ during the adsorption, and the strongest electron‐rich region appears near the Al atom, which means that Al_2_O_3_ effectively regulated the redistribution of space charge (Figure S19, Supporting Information).^[^ ^32^ ^]^

Finite element simulation (FES) was conducted to validate the hypothesis of the “nanopump‐like” effect in Al_2_O_3_/i‐Ti_3_C_2_T_x_ (Text, Supporting Information). Previously reported that momentum can be transferred from free electrons to water molecules in a nanomaterial chamber by applying a voltage, thus promoting directional movement.^[^ ^33^ ^]^ Similarly, the narrow interlayer of Al_2_O_3_/i‐Ti_3_C_2_T_x_ provided an ideal nano‐space to promote the hydrated ions of F^−^ to interact with electrons under an electric field, thereby rapidly passing through the wide interspace to sufficient active sites. Two different 2D models of non‐uniform lamellae with NPs loaded (Al_2_O_3_/i‐Ti_3_C_2_T_x_) and uniform lamellae (Ti_3_C_2_T_x_) were constructed in Figure 6i. At any given time, the F^−^ concentration in Al_2_O_3_/i‐Ti_3_C_2_T_x_ model was much higher, reflecting the stronger F^−^ capture ability. Especially near the NPs, the F^−^ concentration reached up to 0.445 mg m^−3^ (about three times higher than that in Ti_3_C_2_T_x_ model) (Figure S20, Supporting Information). Furthermore, under the same time gradient, the change of F^−^ concentration in Al_2_O_3_/Ti_3_C_2_ model is more obvious (the slope of the curve in Figure S20, Supporting Information), indicating its faster defluoridation speed.^[^ ^34^ ^]^ The FES further strengthened our speculation of “nanopump‐like” effect, that is, hydrated fluoride ions will be transported at a faster rate in the narrow interlayer, thus greatly improving the mass transfer rate of the material.

## 3 Conclusion

Overall, we have successfully demonstrated a strategy for simultaneous improvement of the physical structure and interfacial chemistry of MXene, and showed remarkable performance in electrochemical defluoridation. The wedge‐like microstructure of Al_2_O_3_/i‐Ti_3_C_2_T_x_ was constructed through the integrated strategy of micro‐regulation interlayer space and in situ modification of MXenes. The optimized etching degree precisely regulates the sub‐nanoscale interlayer space of Ti_3_C_2_T_x_, helping to adapt to the efficient capture of small‐size ions. The effective use of A‐layer elements significantly improves the selective site of Ti_3_C_2_T_x_ for trapping F^−^, and neutralizes the negative charge on its surface which is not conducive to defluoridation during the in situ derivatization process. Meanwhile, its wedge‐like microstructure with uneven layers of spacing creates a “nanopump‐like” effect, which exhibited a surprising defluoridation rate. The interfacial conversion‐intercalation pseudocapacitive capture mechanism of fluorine based on Ti_3_C_2_T_x_ and Al_2_O_3_ was revealed. Finally, Al_2_O_3_/i‐Ti_3_C_2_T_x_ accelerated the rapid migration of free electrons and F^−^ by finite element simulation. This work achieved a breakthrough for extending the pseudocapacitive F^−^ capture limit of MXenes, which also provided a universal idea for efficient capture of varisized ions of intercalation materials.

## 4 Experimental Section

### Synthesis of Ti_3_C_2_T_x_

Ti_3_C_2_T_x_ was obtained by the HF etching method. 2.0 g commercial Ti_3_AlC_2_ (400 mesh, 11 Technology Co., Ltd., Jilin Province, China) was tardily added into 40mL HF solution (40% concentration, Aladdin Industrial) at 25°C, then the mixing system was continuously reacted at 400 rpm for 24h. The black sediments were collected by centrifugation and repeatedly washed with HCl solution (1 mol L^−1^, Aladdin Industrial) and deionized (DI) water in turn until the suspension pH was neutral.

### Synthesis of i‐Ti_3_C_2_T_x_

i‐Ti_3_C_2_T_x_ of different etching degrees was obtained by adjusting the HF preparation method of Ti_3_C_2_T_x_. To be specific, 2.0 g commercial Ti_3_AlC_2_ was severally added into 20mL HF solution (24.5% concentration of 12.25%, 19.6%, 24.5%, 29.4%, and 36.75%) at 25°C, then the mixing system was continuously reacted at 400 rpm for 15h. The solid was repeatedly washed with HCl solution (1 mol L^−1^, Aladdin Industrial) and deionized (DI) water in turn until the suspension pH was neutral. The obtained precipitation was freeze‐dried for 12h, and the powder samples of i‐Ti_3_C_2_T_x_‐12.25, i‐Ti_3_C_2_T_x_‐19.6, i‐Ti_3_C_2_T_x_‐24.5, i‐Ti_3_C_2_T_x_‐29.4, and i‐Ti_3_C_2_T_x_‐36.75 were finally obtained.

### Synthesis of Al_2_O_3_/i‐Ti_3_C_2_T_x_

Al_2_O_3_/i‐Ti_3_C_2_T_x_ powder was obtained by annealing the above products, that is, kept at 400 °C for 4h in Ar atmosphere, and finally dropped to room temperature naturally.

### Material Characterization

The morphology was characterized by SEM (Hitachi S‐4800, Japan), TEM (JEM‐2100F, Japan), and HRTEM (JEM‐2010F, Japan). The crystal structure was analyzed by XRD (Bruker D8 Advance, Germany) and XPS (XSAM 800 spectrometer, Kratos Co., UK). The element species and distribution were examined using an energy‐dispersive X‐ray spectroscope (EDX) in conjunction with the SEM instrument. Fourier‐transform infrared (FTIR) spectrum was tested by using a Nicolet iS20 FTIR spectrometer (Thermo Fisher, USA).

### Electrochemical Measurements

In the three‐electrode system, the working electrode (WE) was composed of i‐Ti_3_C_2_T_x_ and Al_2_O_3_/i‐Ti_3_C_2_T_x_ (2 µL electrode material slurry was dropped onto WE), counter electrode (CE) was Pt mesh, reference electrode (RE) was Ag/AgCl and the electrolyte was a 0.5 M NaF aqueous solution. A CHI660D electrochemical workstation (Shanghai CH Instruments Co., China) was used for electrochemical impedance spectroscopy (EIS), constant current charge/discharge (GCD), and cyclic voltammetry (CV) tests.

### Defluoridation Measurements

The active materials (i‐Ti_3_C_2_, Al_2_O_3_/Ti_3_C_2_, and active carbon), carbon black (Sinopharm Chemical Reagent Co., Ltd), and polyvinylidene (PVDF, Shanghai Macklin Biochemical Co., Ltd.) were mixed at a mass ratio of 8:1:1 and stirred for 1h to obtain a homogenous mixture. Then slowly added N‐methyl‐2‐pyrrolidone (MNP, Aladdin Industrial) to the mixture until it was a slurry, and then stirred for 12h to make the slurry uniform. The resulting uniform slurry was applied on the carbon paper and dried at 60°C for 12h to obtain the electrode with a size of 4 × 4 cm^2^ and a thickness of 75 mm. A constant‐voltage and batch‐mode system consisting of a CDI cell, a constant voltage power supply device (LAND battery testing system), a conductivity meter (Mettler Toledo S230, Switzerland), a peristaltic pump (BT100‐2J, LONGER, China) and NaF solution tank, was tested for defluoridation. The membrane CDI device was similar to the previously reported device, which is shown in Figure S13 (Supporting Information). Before each defluoridation test, the electrodes were washed in DI water and NaF solution successively for 12h. The defluoridation efficiency of F^−^ solution with different initial concentrations (10 and 100 mg L^−1^) and different voltage (1.0–1.6 V) was studied. In addition, a pH meter (Mettler Toledo S700‐K, Switzerland) was used to continuously monitor pH fluctuations during charge and discharge. Considering the complexity of the water sample in the actual environment, the influence of complex ions in feed water on the defluoridation performance was explored, which were respectively: 150 mg L^−1^ of Cl^−^, CO_3_^2−^, SO_4_^2−^, and NO_3_^−^ mixed with 100 mg L^−1^ F^−^. The F^−^ concentration was measured by a fluoride ion selective electrode (Multi 3320, ISE).

## Conflict of Interest

The authors declare no conflict of interest.

## Supporting information



Supporting Information

## Data Availability

The data that support the findings of this study are available from the corresponding author upon reasonable request.
