# Supplementary material for: Wedge‐Like Microstructure of Al2O3/i‐Ti3C2Tx Electrode with “Nano‐Pumping” Effect for Boosting Ion Diffusion and Electrochemical Defluoridation
Source: Adv Sci (Weinh). 2024 Nov 22;12(3):2411659. doi: 10.1002/advs.202411659 (PMC11744565; doi:10.1002/advs.202411659)
Supplement: Supplementary file 1 — Supporting Information [file ADVS-12-2411659-s001.docx]

Supporting Information

**Wedge-like Microstructure of Al_2_O_3_ /Ti_3_C_2_T_x_ Electrode with “Nano-pumping” Effect for Boosting Ion Diffusion and Electrochemical Defluorination**

Junce Wang^1,2^, Jinfeng Chen^2^, Ningning Liu^2^, Jingjing Lei^2^, Hong-wen Gao^2^, Fei Yu^1,3*^, Fanghui Pan^1^, Jie Ma^1,2*^

1 School of Civil Engineering, Kashi University, Kashi 844000, P. R. China

2 Research Center for Environmental Functional Materials, State Key Laboratory of Pollution Control and Resource Reuse, College of Environmental Science and Engineering, Tongji University, Shanghai, 200092, P. R. China

3 College of Oceanography and Ecological Science, Shanghai Ocean University, No 999, Huchenghuan Road, Shanghai, 201306, P. R. China

Corresponding author:

Fei Yu, E-mail:fyu@vip.163.com

Jie Ma, E-mail: jma@tongji.edu.cn, Tel:+86-021-65981926

**Supplementary Information Text**

**Calculation method and equations**

The F^-^ adsorption capacity (FAC, mg g^-1^) was calculated using Equation (1):

$$\begin{aligned} FAC= \frac{\left( C_{i}-C_{t} \right)\times V}{m}\#\left( 1 \right) \end{aligned}$$

where C_i_ (mg L^−1^) and C_t_ (mg L^−1^) is the initial and treated concentration of sewage respectively, m (g) is the weight of the Al_2_O_3_/Ti_3_C_2_ electrode, and V (L) is the volume of sewage.

The fluorine removal rate (FAR, mg g^-1^ min^-1^) was calculated using Equation (2):

$$\begin{aligned} FAR= \frac{\mathrm{FAC}}{\Delta T}\times60\#\left( 2 \right) \end{aligned}$$

where ΔT (s) is the time interval from time i to time t.

The energy consumption (EC, kWh kg_NaF_^-1^) was calculated using Equation (3):

$$\begin{aligned} EC= \frac{v\times\int\mathrm{idt}}{3.6\times\left( C_{i}-C_{t} \right)\times V}\#\left( 3 \right) \end{aligned}$$

where v (V) is the voltage applied during the fluorine removal process, i (mA) is the current required for the defluorination process, and t (s) is the time of the defluorination process.

The charge efficiency (Λ) was calculated using Equation (4):

$$\begin{aligned} \Lambda= \frac{\Gamma\times F}{\Sigma}\#\left( 4 \right) \end{aligned}$$

where Γ (mol g^-1^) is defluorination capacity, F is the Faraday constant (96485 C mol^-1^), and ∑ (Charge, C g^-1^) is obtained by integrating current.

**Finite element simulation**

In this section, A simplified 2D method is used to simulate the mass transfer reaction in 2D profile space. The simulation under different structure design and different active site design is simulated. According to the morphologies of Al_2_O_3_/Ti_3_C_2_ obtained in the SEM and TEM images, we have rationally designed the plane models of lamellar axial section, uniform and ununiform interlayer space, for finite element simulations. The dimensions of the two models are 3300 × 4200 nm^2^, and the uniform layer spacing is about 200 nm. In the wedge-shaped inhomogeneous layer space model, the narrow spacing is about 100-150 nm, the wide spacing is about 200-300 nm, and the particle size is 40-50 nm. Based on the defluorination properties of the NaF solution, we provided the model with a salt concentration of 10 ppm at the inlet. Assuming the F^-^ concentration around two materials from scratch, and its simulation to put a 1.4 V voltage of 10 mg m^-3^ F^-^ concentration of environment is studied.

In terms of calculation, the dilute material transfer interface is satisfied as a whole, and mass transfer is simulated by diffusion Equation (5):

$$\begin{aligned} \nabla\cdot\left( -D\nabla c \right)=0\#\left( 5 \right) \end{aligned}$$

where c (mol m^-3^) is the concentration of NaF solution, D (m^2^ s^-1^) is diffusivity, and the whole follows Fick's law of diffusion.

There is a persistent consumption reaction on the surface of the particles is simulated by Equation (6):

$$\begin{aligned} k_{S}=A\cdot c\#\left( 6 \right) \end{aligned}$$

where A represents the surface reaction frequency.

The outer boundary concentration and the initial relative concentration are set as 0 mol L^-1^. The diffusion coefficient is set as 5e^-10^ (m^2^ s^-1^); The surface reaction frequency of the particles is 5e^-6^ m s^-1^.

**Supplementary Figures**


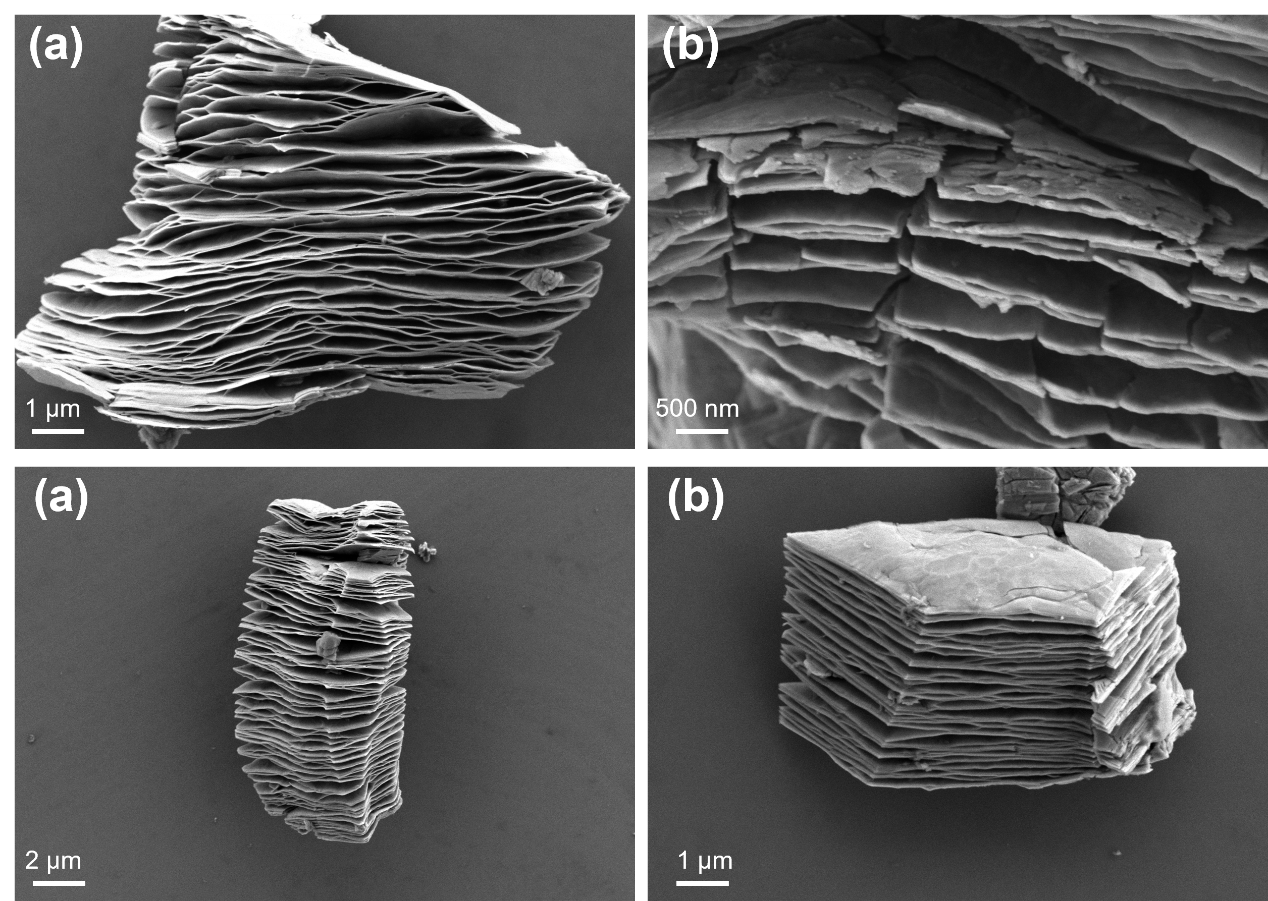


**Figure. S1.** SEM images of (a) i-Ti_3_C_2_T_x_-36.75, (b) i-Ti_3_C_2_T_x_-36.75 after calcination, (c) Ti_3_C_2_T_x_ and (d) Ti_3_C_2_T_x_ after calcination.

The SEM image of i-Ti_3_C_2_T_x_-36.75 etched with 20mL 36.75% HF in 15h (**Figure S1a**) showed a highly layered accordion-like structure, and still maintained the morphology without NPs after calcination (**Figure S1b**). The SEM image of Ti_3_C_2_T_x_ etched with 40mL 40% HF in 24h (**Figure S1c**) showed a complete accordion-like structure, and still maintained the morphology without NPs after calcination (**Figure S1d**).


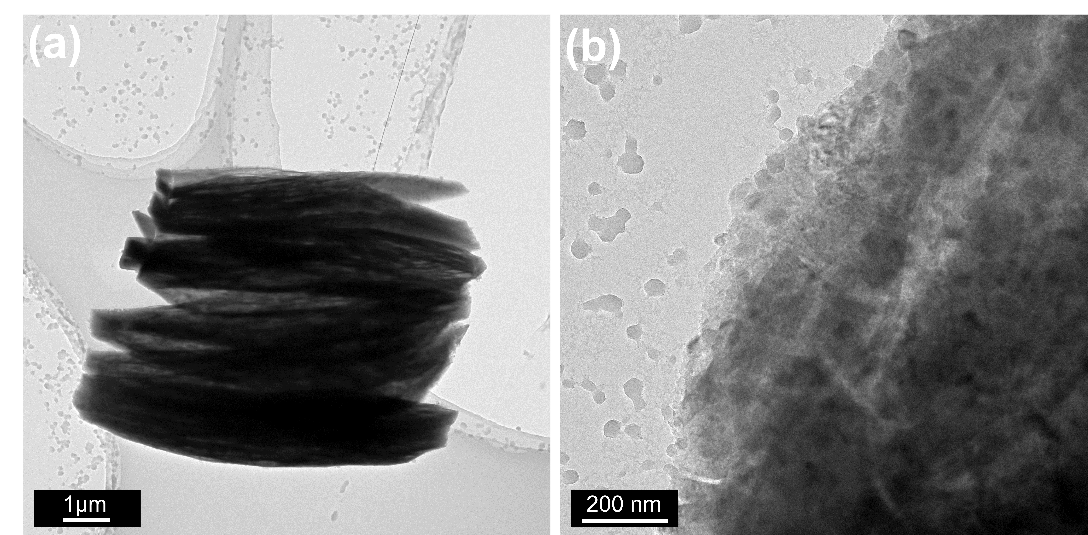


**Figure. S2.** TEM images of (a) i-Ti_3_C_2_T_x_-24.5, (b) Al_2_O_3_/i-Ti_3_C_2_T_x_-24.5.


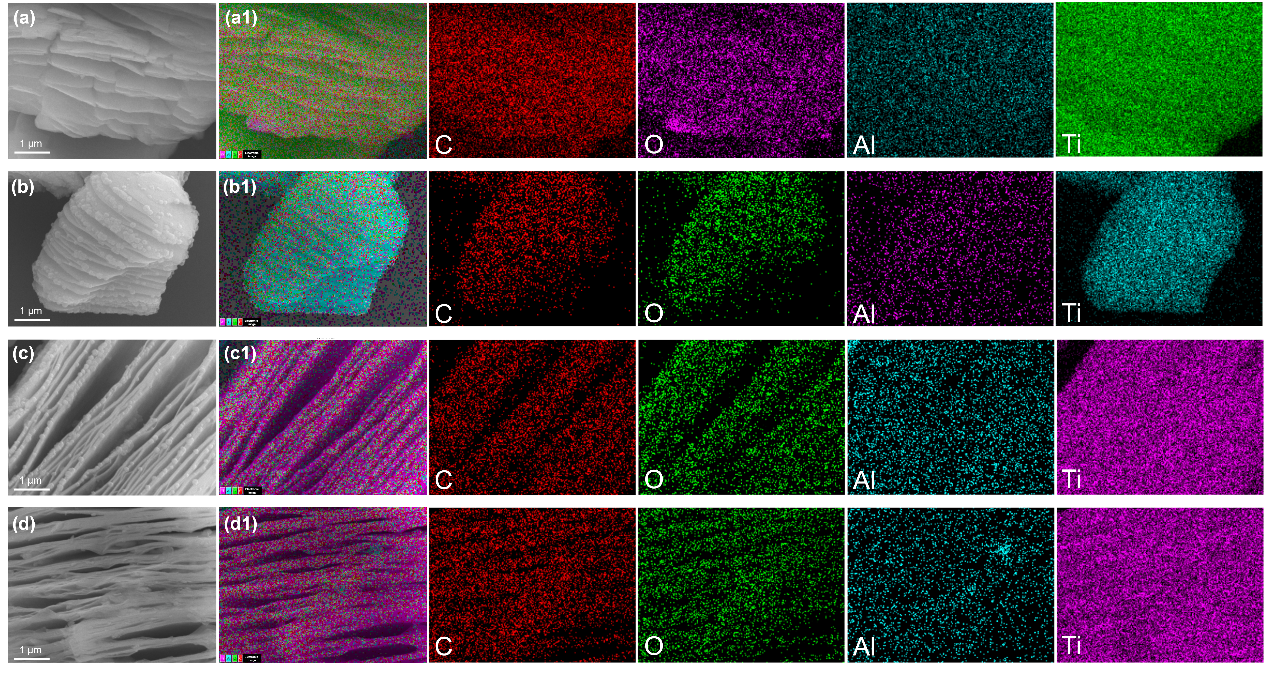


**Figure S3.** EDS element mapping (first column) and Layered (second column) images of (a, a1) i-Ti_3_C_2_T_x_-36.75 after calcination, (b, b1) Al_2_O_3_/i-Ti_3_C_2_T_x_-29.4, (c, c1) Al_2_O_3_/i-Ti_3_C_2_T_x_-19.6 and (d, d1) Al_2_O_3_/i-Ti_3_C_2_T_x_-12.25.


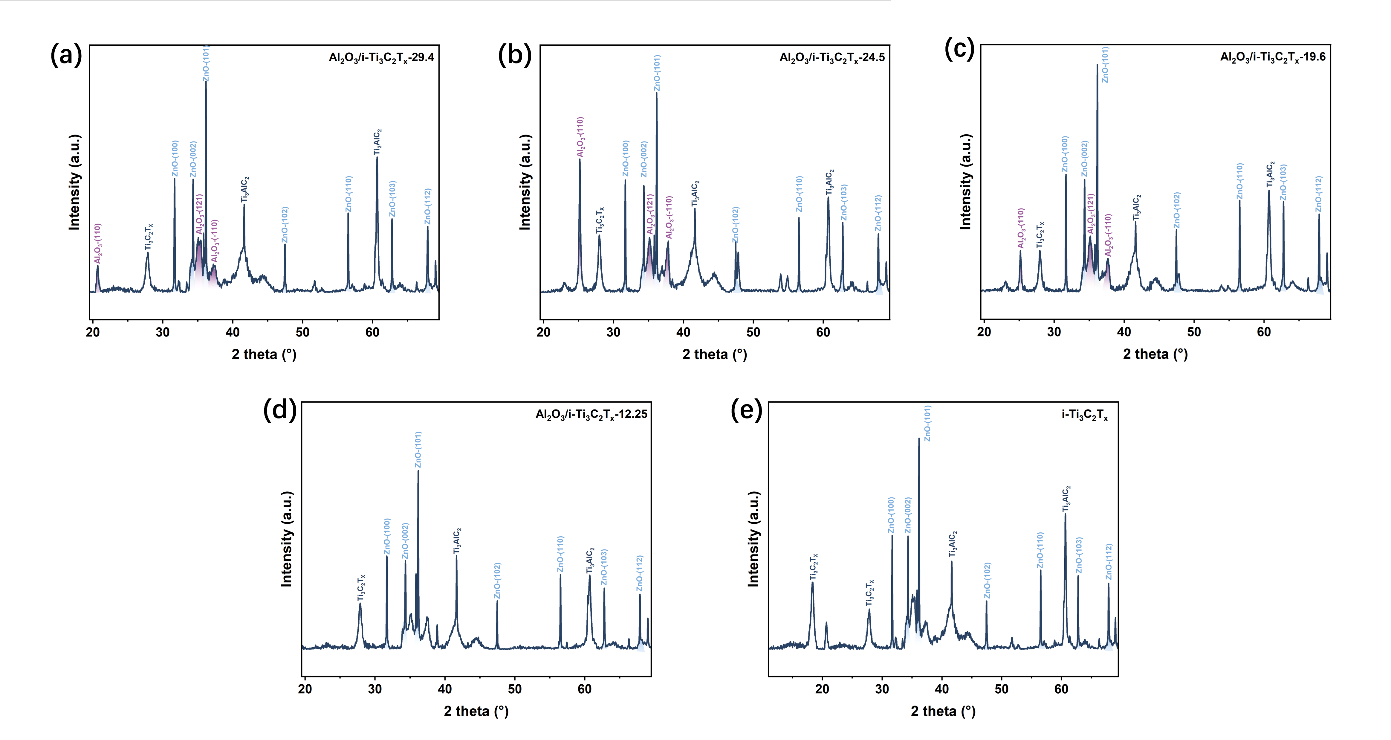


**Figure S4.** Internal standard XRD pattern of (a) Al_2_O_3_/i-Ti_3_C_2_T_x_-29.4, (b) Al_2_O_3_/i-Ti_3_C_2_T_x_-24.5, (c) Al_2_O_3_/i-Ti_3_C_2_T_x_-19.6, (d) Al_2_O_3_/i-Ti_3_C_2_T_x_-12.25, and (e) i-Ti_3_C_2_T_x_.

During XRD measurement of all samples, ZnO with 10% content was added, where ZnO as internal standard was used for quantitative analysis of Al_2_O_3_ phase content in XRD by Jade software. The analysis results show that Al_2_O_3_/i-Ti_3_C_2_T_x_-12.25 and i-Ti_3_C_2_T_x_ were 0% (no Al_2_O_3_ phase was identified in XRD pattern), and that of Al_2_O_3_/i-Ti_3_C_2_T_x_-29.4/24.5/19.6 were 5.0%, 6.9% and 3.8%, respectively.


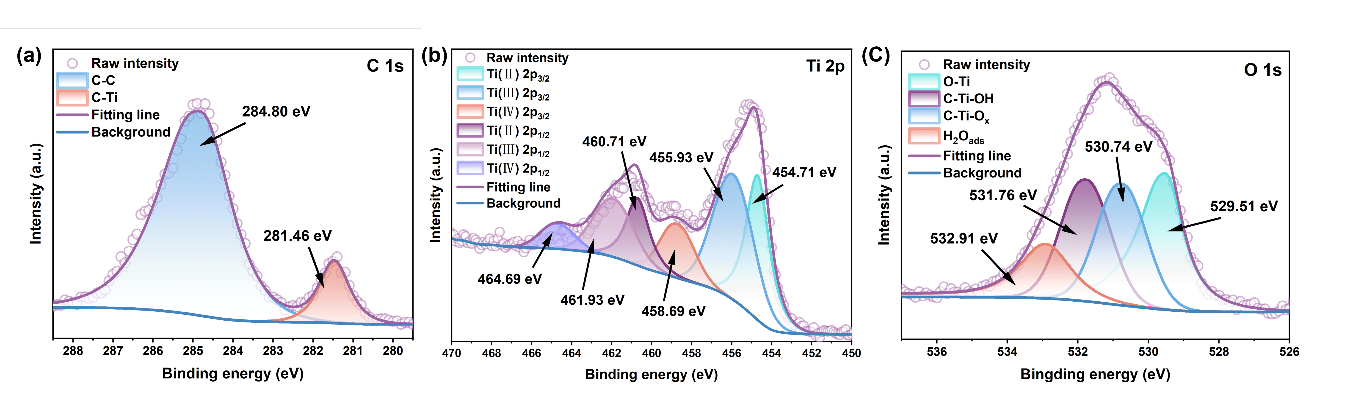


**Figure S5.** High-resolution XPS spectra of (a) C 1s, (b) Ti 2p, and (c) O 1s of i-Ti_3_C_2_T_x_.

In **Figure S5a,** C-C (284.80 eV) and C-Ti (281.46 eV) bonds proved the samples retained the complete structure of Ti_3_C_2_T_x._ Peaks at 454.71 eV, 455.91 eV, and 458.69 eV were indexed as Ti (Ⅱ), Ti (Ⅲ), and Ti (Ⅳ) (**Figure S5b**), corresponding to carbon bonded titanium atoms with terminals and oxidized titanium (C-Ti-F, C-Ti-O, and O-Ti-O). Notably, the peak strength of O-Ti-O was increased compared with Al_2_O_3_/i-Ti_3_C_2_T_x_-24.5, which may be due to the fact that i-Ti_3_C_2_T_x_ was more prone to oxidation of “active” titanium atoms after long-term storage. Four main peaks at 529.51 eV, 530.74 eV, 531.76 eV, and 532.91 eV in O 1s spectrum (**Figure S5c**) were related to functionalized Ti-O_x_, C-Ti-O_x_, C-Ti-OH, and absorbed H_2_O, respectively.


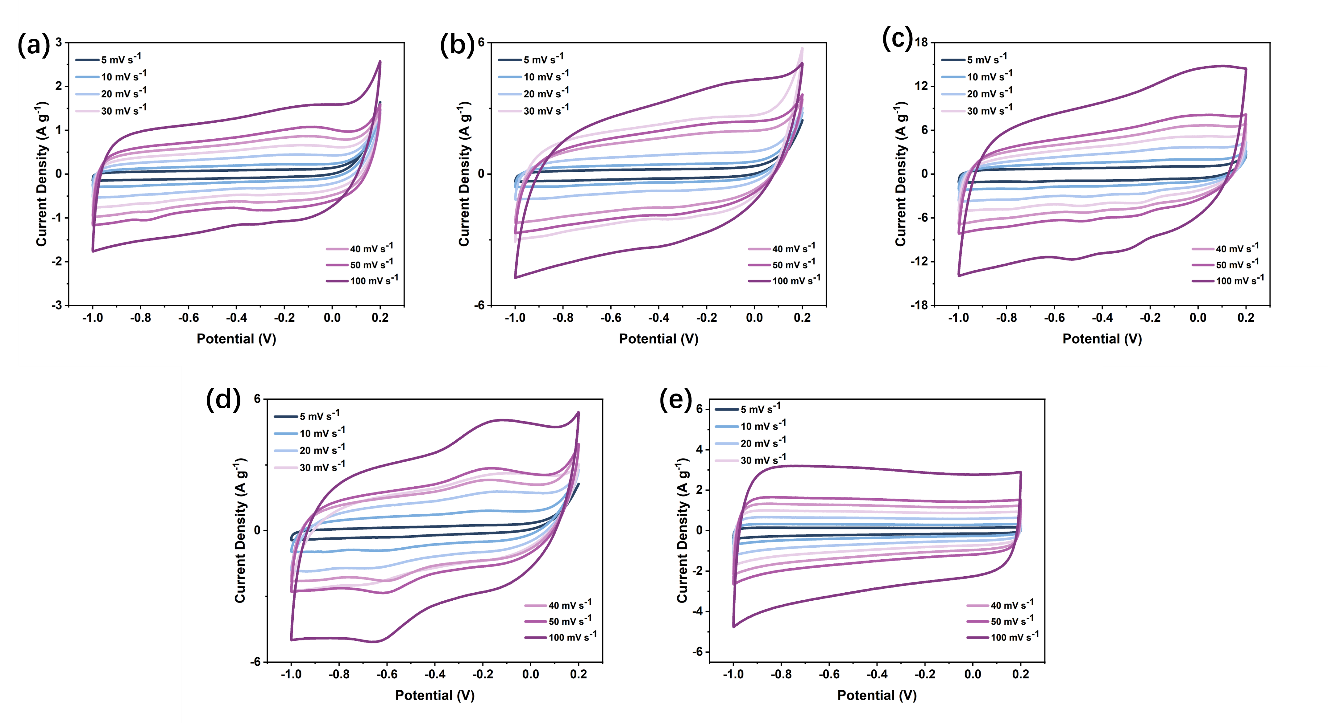


**Figure S6.** The CV curves of (a) Al_2_O_3_/i-Ti_3_C_2_T_x_-12.25, (b) Al_2_O_3_/i-Ti_3_C_2_T_x_-19.6, (c) Al_2_O_3_/i-Ti_3_C_2_T_x_-24.5, (d) Al_2_O_3_/i-Ti_3_C_2_T_x_-29.4, and (e) i-Ti_3_C_2_T_x_. at different rates (5 mV s^-1^, 10 mV s^-1^, 20 mV s^-1^, 30 mV s^-1^, 40 mV s^-1^, 50 mV s^-1^, and 60 mV s^-1^).

As shown in **Figure S6,** the CV curves of all samples increased with the increase of scan rates, and Al_2_O_3_/i-Ti_3_C_2_T_x_-24.5 was much higher than other samples. Importantly, the CV images of Al_2_O_3_/i-Ti_3_C_2_T_x_-12.25 and i-Ti_3_C_2_T_x_ show a squarer rectangle and a more obvious polarization phenomenon, while the samples containing Al_2_O_3_ showed a distorted rectangle-like shape, which can be attributed to Al_2_O_3_ supplementing the electrochemical fluorine storage potential of the active site.


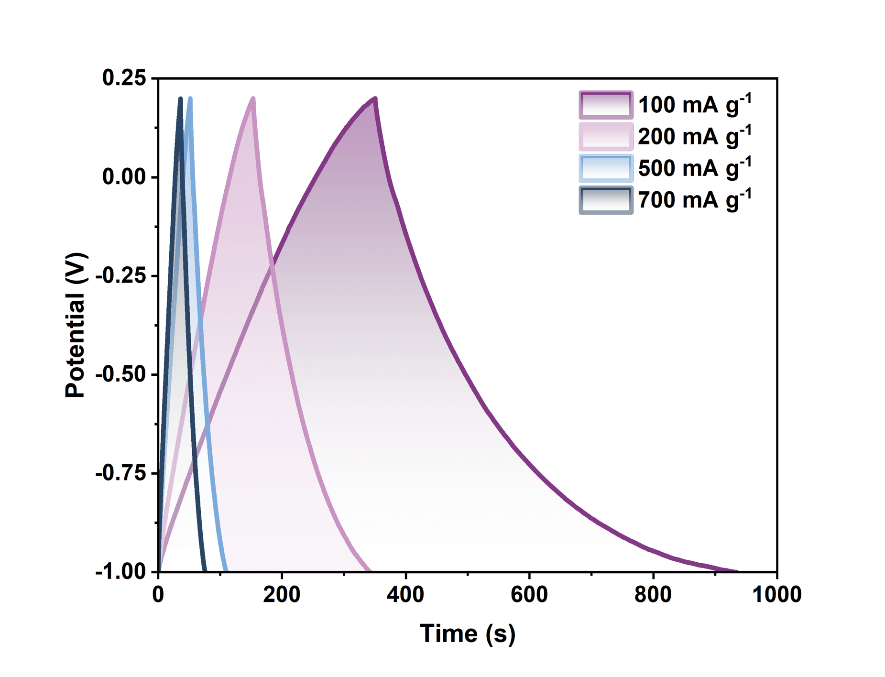


**Figure S7.** The GCD curves of Al_2_O_3_/i-Ti_3_C_2_T_x_-24.5 at current density (100 mA g^-1^, 200 mA g^-1^, 500 mA g^-1^, and 700 mA g^-1^).


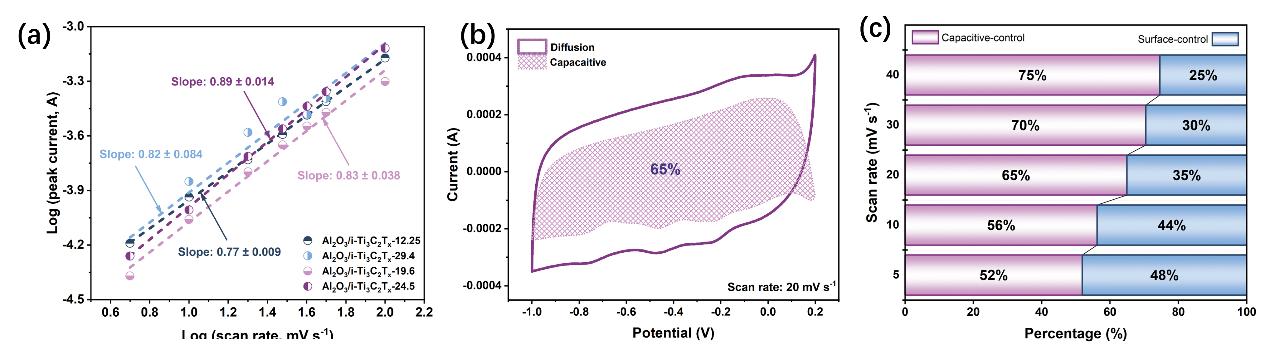


**Figure S8.** (a) Power law relationship between peak current of Al_2_O_3_/i-Ti_3_C_2_T_x_-29.4/24.5/19.6/12.25, (b) CV curve at 20 mVs-1 where the shaded area represented capacitive contribution and (c) capacitive and diffusion contribution ratio at different scan rates of Al_2_O_3_/i-Ti_3_C_2_T_x_-24.5.

The *b* value of Al_2_O_3_/Ti_3_C_2_ at various voltages ranged from 0.77 to 0.85, which reflected its strong pseudocapacitance-dominated behavior during ions storage (**Figure S8a**). In other words, the *b* value close to 1 further validated that Al_2_O_3_/Ti_3_C_2_ exhibited both intercalation-behavior and interfacial conversion-behavior[1]. The capacitance ratio of Al_2_O_3_/Ti_3_C_2_ was as high as 65% at 20 mV s^-1^ (**Figure S8b**). The percentage of capacitance contribution was proportional to scan rates (**Figure S8c**). The increased capacitance contribution at high scan rate reasonably reflected the fast conversion-behavior of Al_2_O_3_ NPs at the interface, while the low scan rate gradually turned to intercalation-behavior played a leading role in the interlayer.


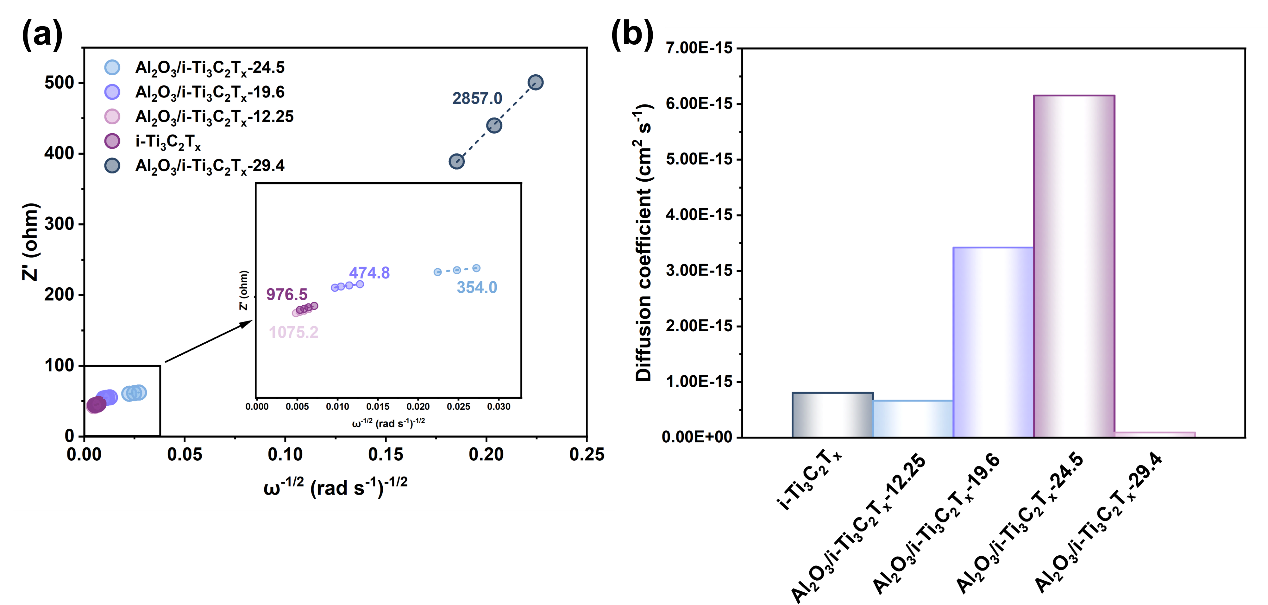


**Figure S9.** (a) Plots of the relationship between Z’ and ω^−1/2^ in the low-frequency region at design potential, (b) diffusion coefficient of samples.


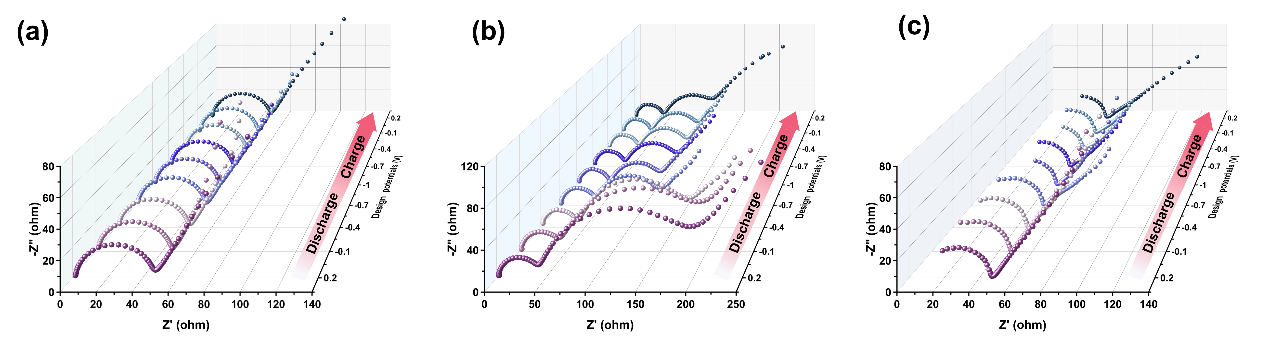


**Figure S10.** (a) Nyquist plots at staircase potentials (-0.8-0.2 V) of (a) Al_2_O_3_/i-Ti_3_C_2_T_x_-24.5, (b) Al_2_O_3_/i-Ti_3_C_2_T_x_-29.4, and (c) Al_2_O_3_/i-Ti_3_C_2_T_x_-12.25


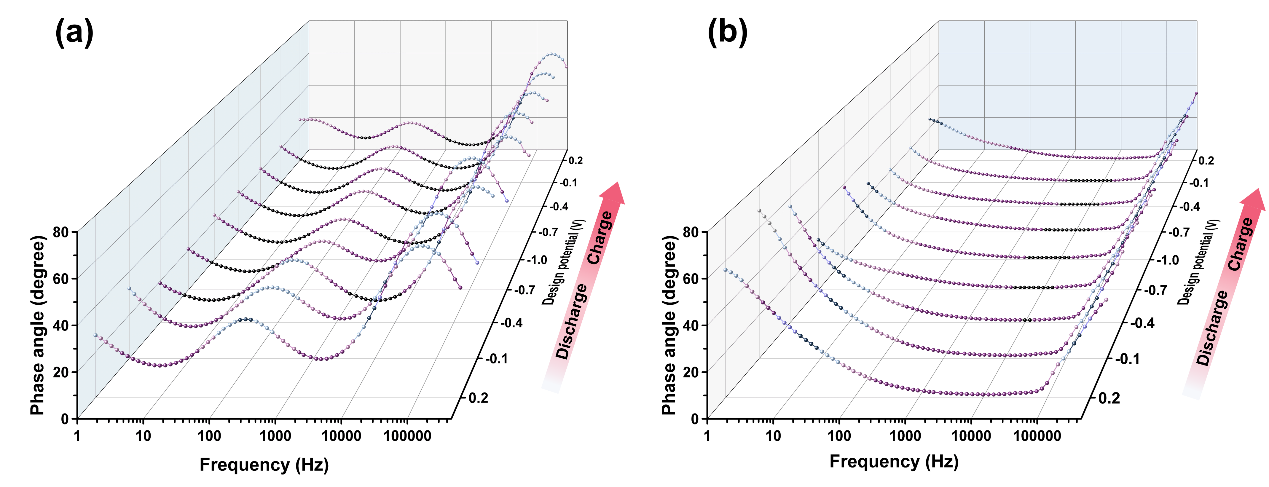


**Figure S11.** Bode plots for (a) Al_2_O_3_/i-Ti_3_C_2_T_x_-29.4 and (b) Al_2_O_3_/i-Ti_3_C_2_T_x_-12.25.


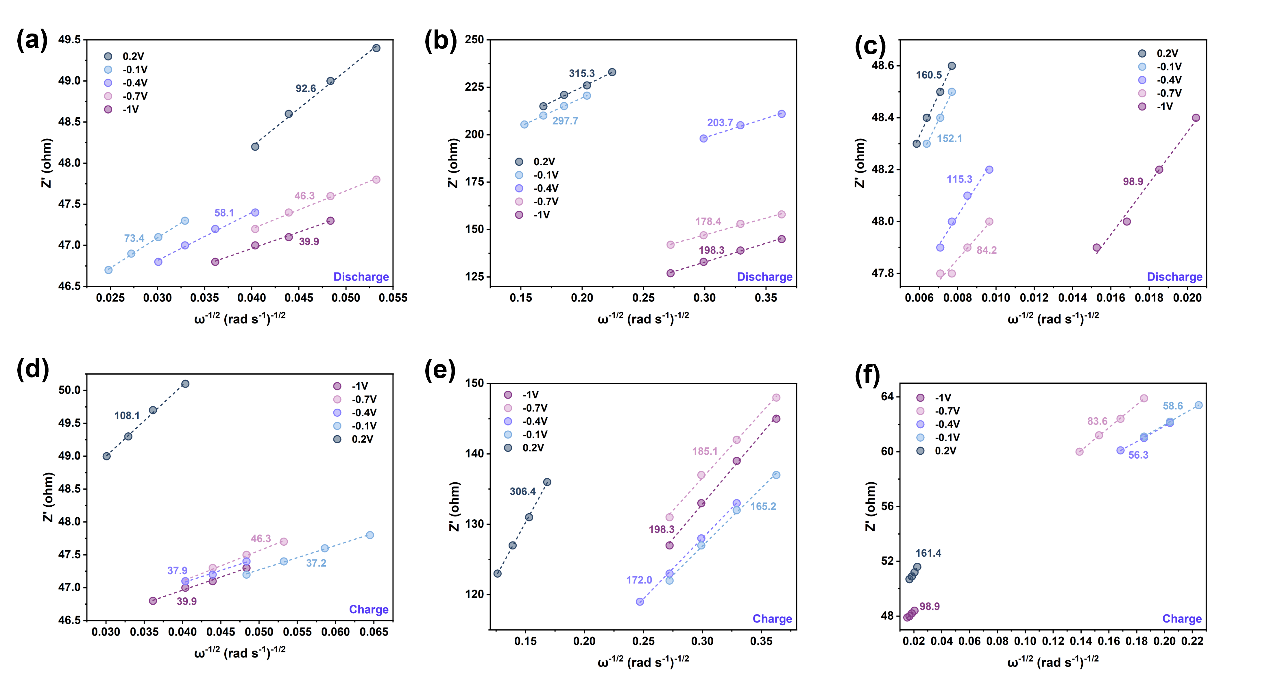


**Figure S12.** Plots of the relationship between Z’ and ω^−1/2^ during charge-discharge of (a,d) Al_2_O_3_/i-Ti_3_C_2_T_x_-24.5, (b,e) Al_2_O_3_/i-Ti_3_C_2_T_x_-29.4, and (c,f) Al_2_O_3_/i-Ti_3_C_2_T_x_-19.6.


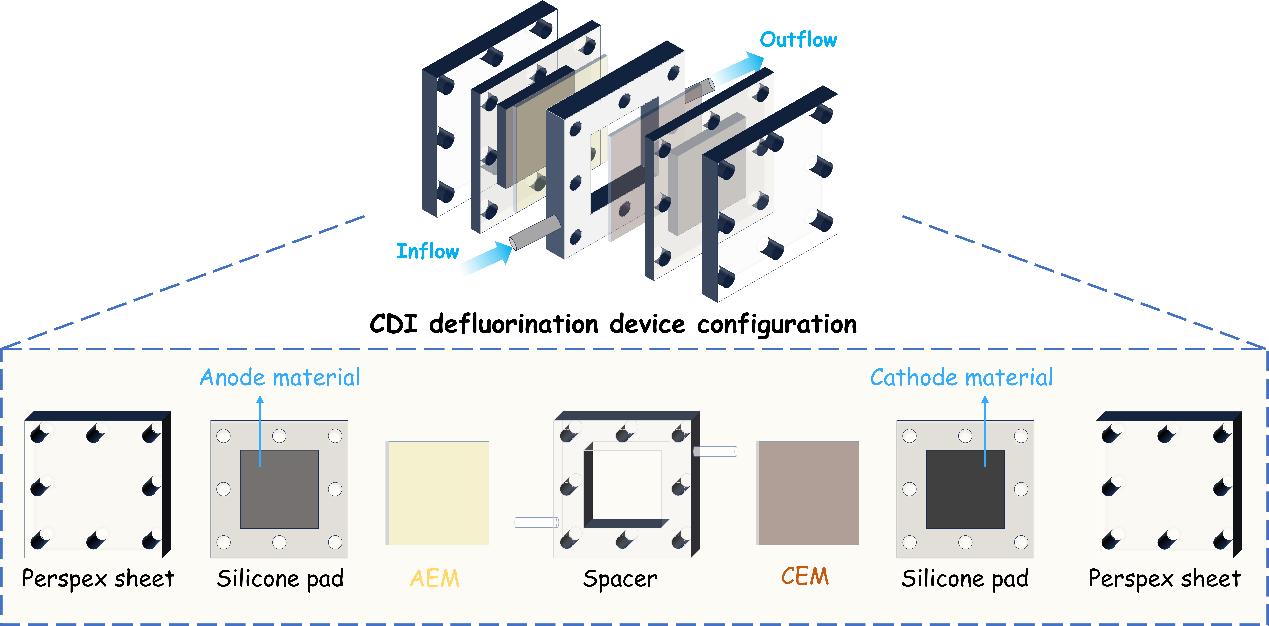


**Fig. S13.** Schematic diagram of the defluorination CDI cell.


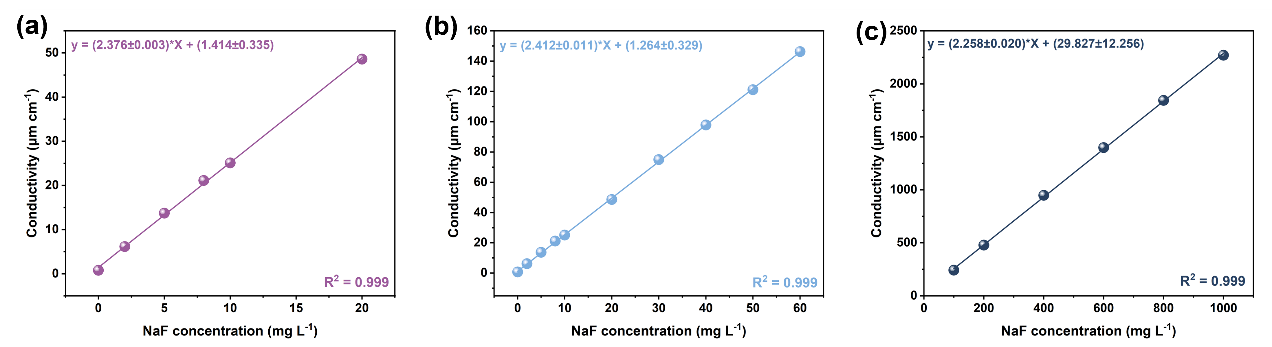


**Figure S14.** Standard curve of conductivity and NaF solution in concentration of (a) 0-20 mg L^-1^, (b) 0-60 mg L^-1^, (c) 100-1000 mg L^-1^.


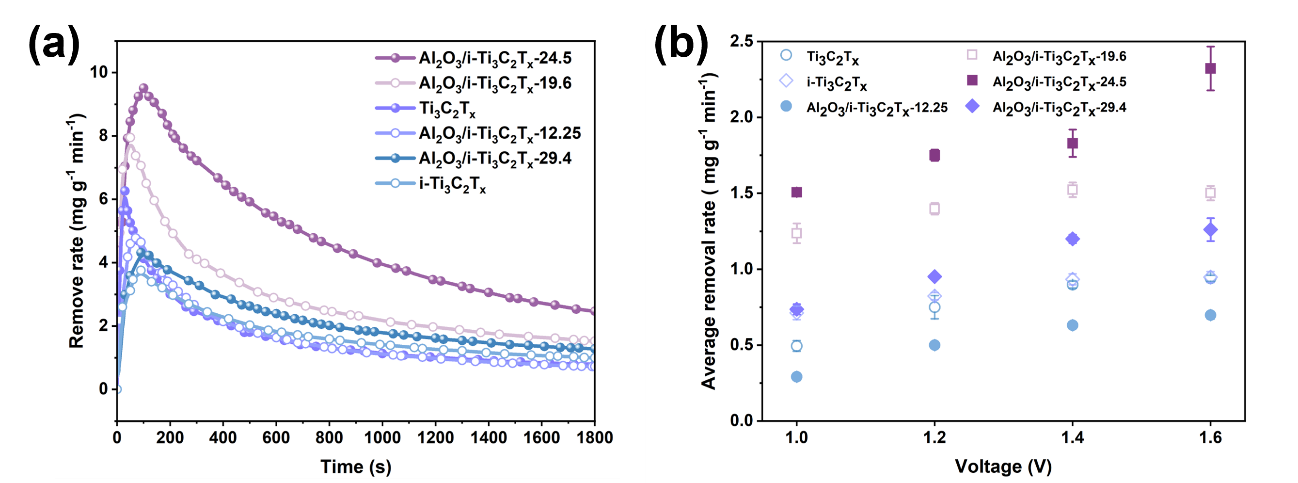


**Figure S15.** (a) Instantaneous defluorination rate during a cycle and (b) average defluorination rate at different voltages of Al_2_O_3_/i-Ti_3_C_2_T_x_-29.4/24.5/19.6/12.25, i-Ti_3_C_2_T_x_, and Ti_3_C_2_T_x_.


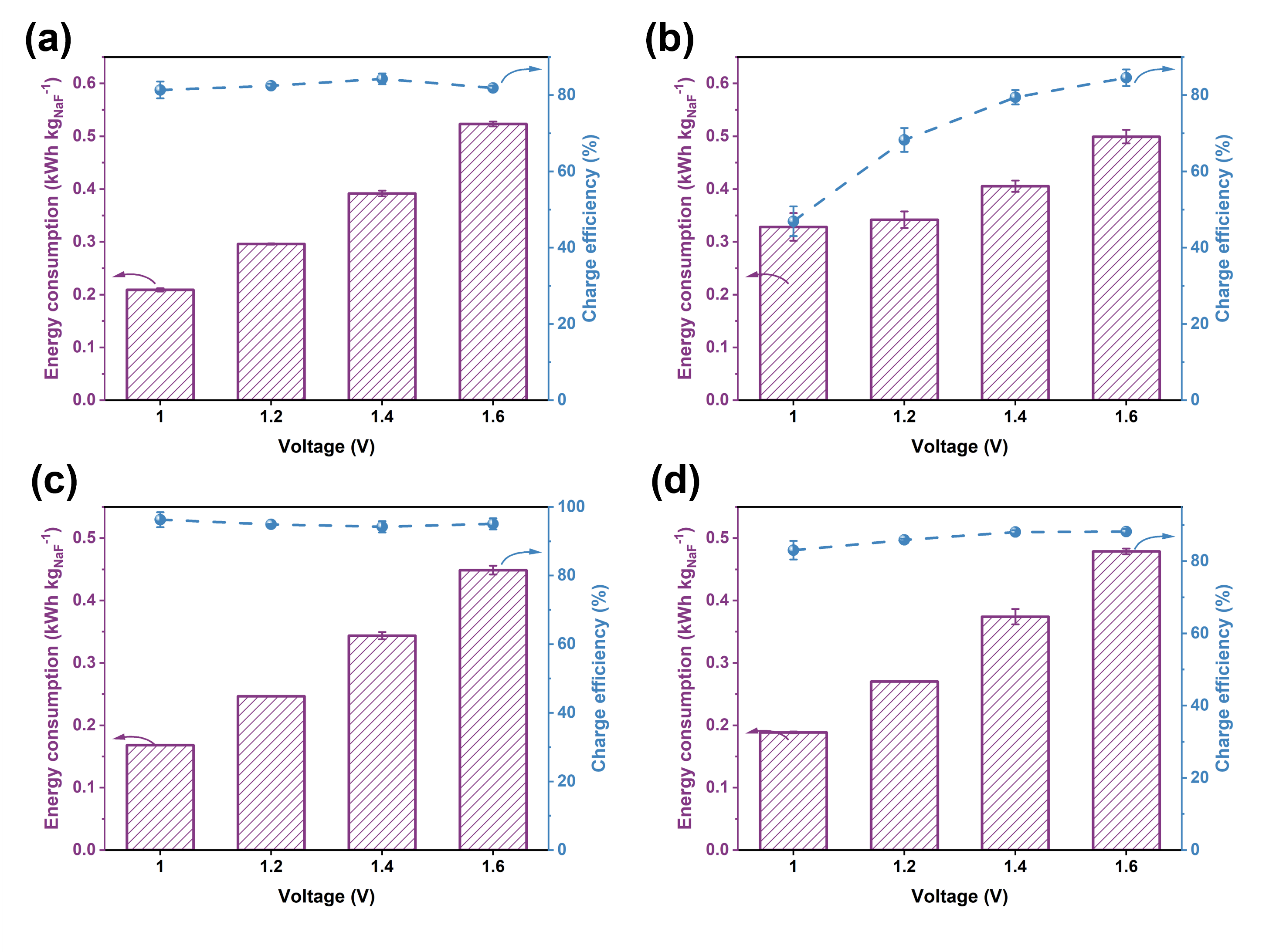


**Figure S16.** Energy consumption and charge efficiency at various voltage (1.0 to 1.4V) of (a) i-Ti_3_C_2_T_x_, (b) Al_2_O_3_/i-Ti_3_C_2_T_x_-12.25, (c) Al_2_O_3_/i-Ti_3_C_2_T_x_-19.6, and (d) Al_2_O_3_/i-Ti_3_C_2_T_x_-29.4.


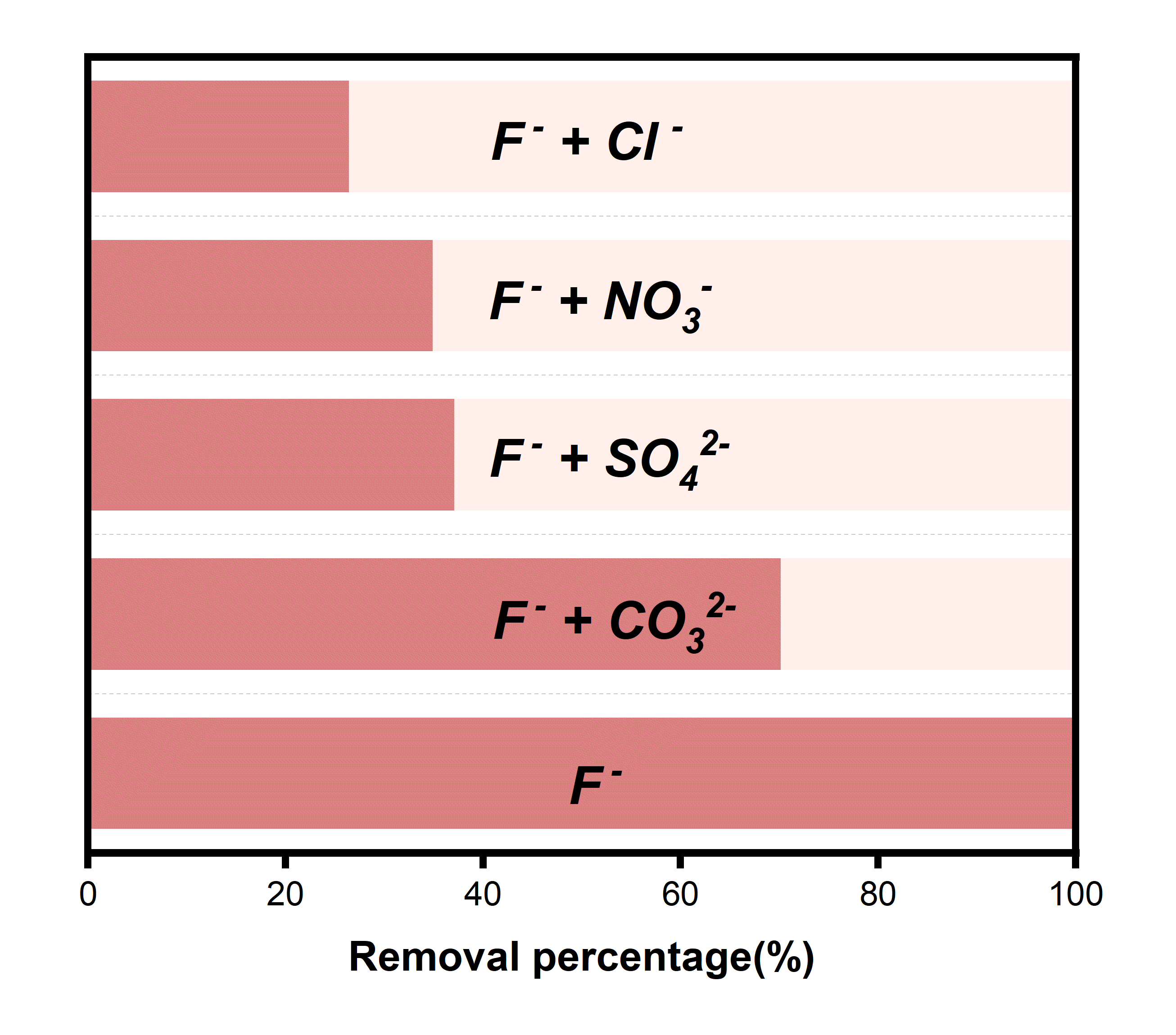


**Figure S17.** Removal percentage of fluorine at interfering ion coexistence.

Simulated real water containing different interfering anions (Cl^-^, NO_3_^-^, SO_4_^2-^ and CO_3_^2-^) was taken to test the anti-interference ability of Al_2_O_3_/i-Ti_3_C_2_T_x_-24.5 electrode. The results showed that Cl^-^ has the greatest interference to F^-^ capture (F^-^ / Cl^-^ = 0.26, F^-^ / NO_3_^-^ = 0.35, F^-^ / SO_4_^2-^ = 0.37, F^-^ / CO_3_^2-^ = 0.70), which can be explained by the fact that Cl^-^ had a relatively small hydration radius to easily embed between MXene layers[2]. Compared with the divalent ions, F^-^ had a smaller dehydrating ion diameter and a stronger affinity with Al_2_O_3_, which helped to compete for active sites at a faster rate[3].

**
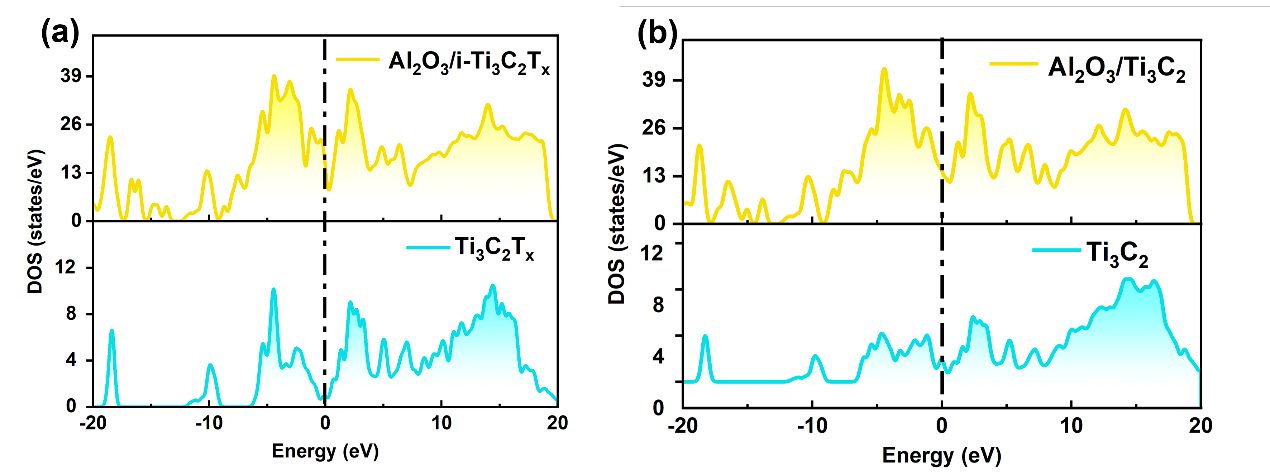
**

**Figure S18.** Calculated DOS of Al_2_O_3_/i-Ti_3_C_2_T_x_ and Ti_3_C_2_T_x_ with the aligned Fermi level (a) before, and (b) after absorbed F.

**
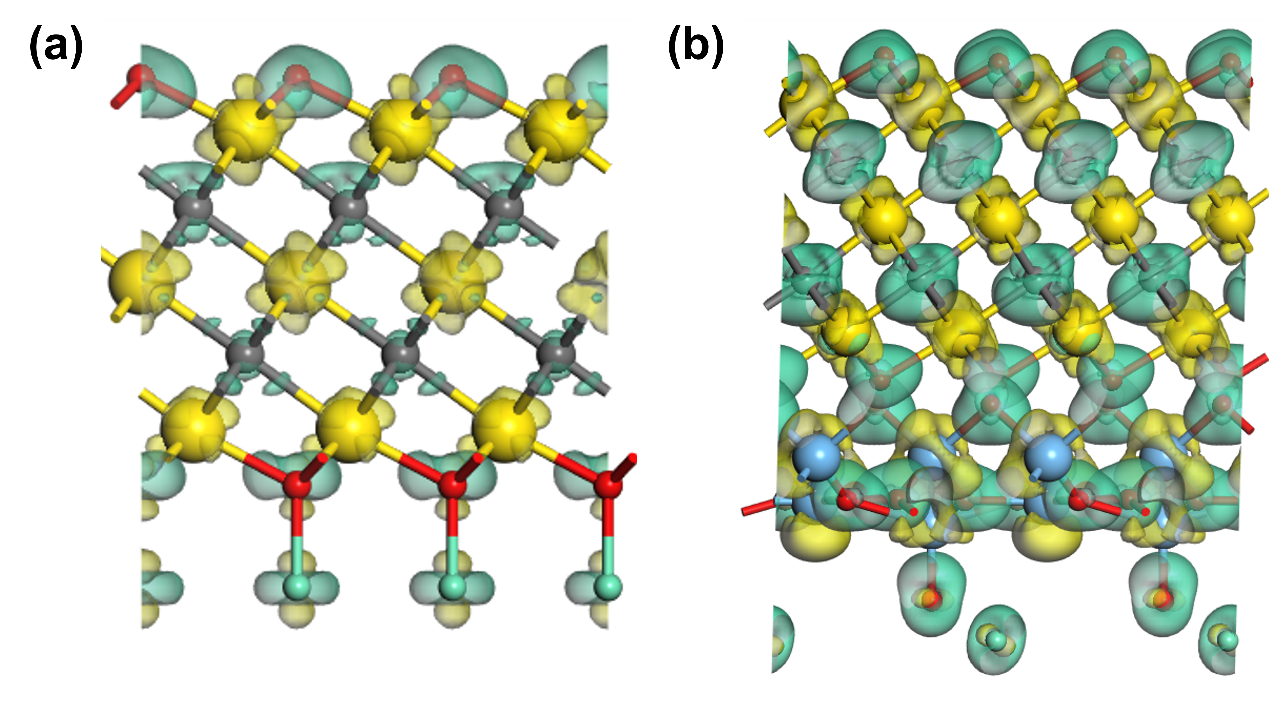
**

**Figure S20.** Charge density differences of (a) Ti_3_C_2_T_x_ and (b) Al_2_O_3_/i-Ti_3_C_2_T_x_ for the capture of F atom.

**
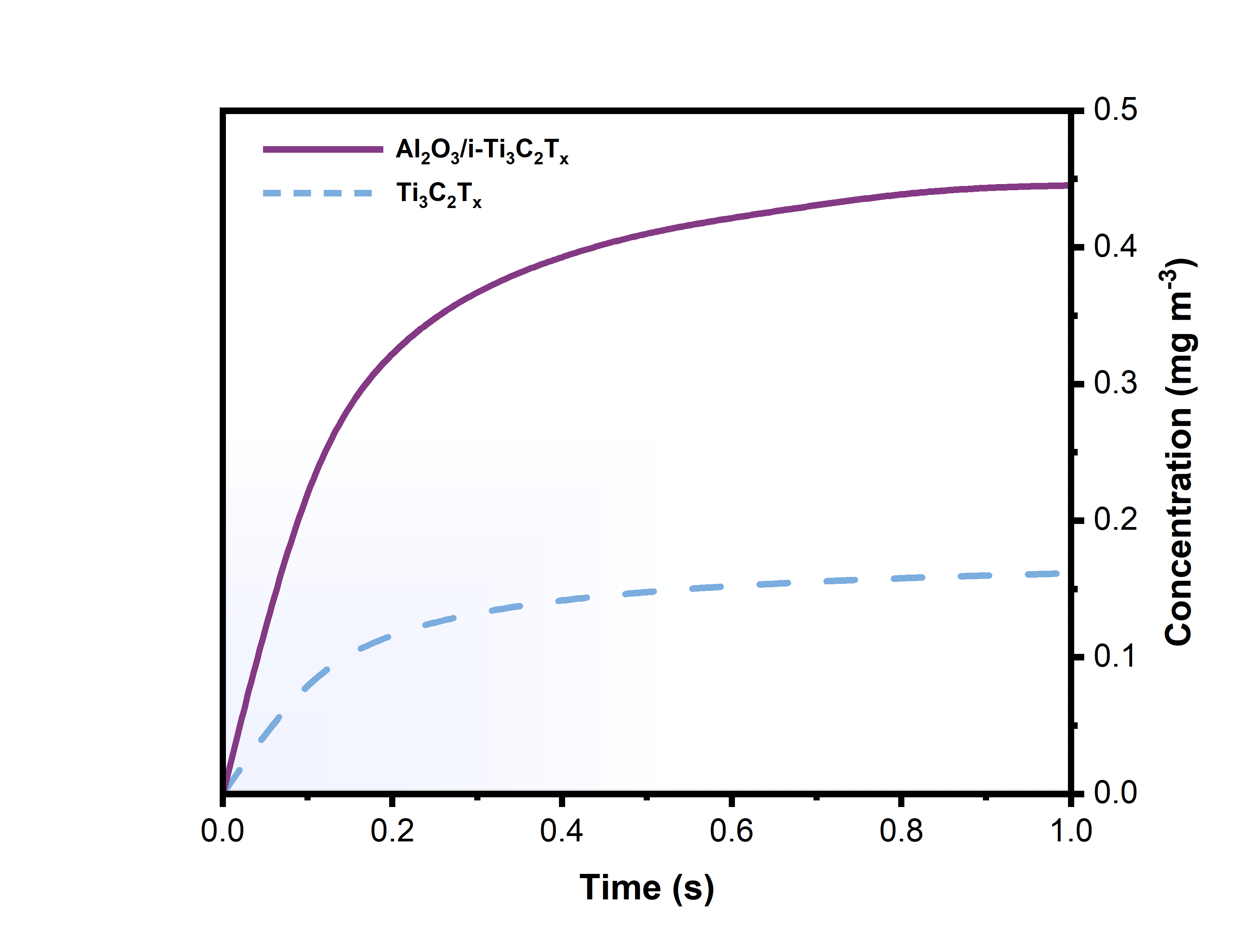
**

**Figure S20.** The trend of F^-^ concentration at the inner boundary of Al_2_O_3_/i-Ti_3_C_2_T_x_ and Ti_3_C_2_T_x_ model with time.

**Supplementary Tables**

**Table S1.** The content (weight percentage) of Al element in each sample was measured by ICP-OES.

| **Samples** | **Al content (wt. %)** | **Average Al content (wt. %)** | **Average error** |
| --- | --- | --- | --- |
| Ti_3_C_2_T_x_ | 2.151 | 2.152 | 3.03747E-05 |
|  | 2.157 |  |  |
|  | 2.149 |  |  |
| Al_2_O_3_/i-Ti_3_C_2_T_x_-29.4 | 4.374 | 4.380 | 4.90977E-05 |
|  | 4.382 |  |  |
|  | 4.386 |  |  |
| Al_2_O_3_/i-Ti_3_C_2_T_x_-24.5 | 7.343 | 7.350 | 5.91076E-05 |
|  | 7.349 |  |  |
|  | 7.357 |  |  |
| Al_2_O_3_/i-Ti_3_C_2_T_x_-19.6 | 12.234 | 12.240 | 4.35928E-05 |
|  | 12.240 |  |  |
|  | 12.245 |  |  |
| Al_2_O_3_/i-Ti_3_C_2_T_x_-12.25 | 14.667 | 14.664 | 2.20758E-05 |
|  | 14.661 |  |  |
|  | 14.664 |  |  |
| Ti_3_AlC_2_ | 16.999 | 17.001 | 1.13254E-05 |
|  | 17.002 |  |  |
|  | 17.002 |  |  |

**Table S2.** The R_s_ and R_ct_ of all samples at open and design (1.4 V) potentials through equivalent circuit fitting.

| **Samples** | **Potential** | **R_s_ (Ω)** | **R_ct_ (****Ω)** |
| --- | --- | --- | --- |
| Al_2_O_3_/i-Ti_3_C_2_T_x_-29.4 | Open state | 1.174 | 34.55 / 174.30 |
|  | Working state | 2.858 | 36.26 |
| Al_2_O_3_/i-Ti_3_C_2_T_x_-24.5 | Open state | 1.961 | 36.60 |
|  | Working state | 1.765 | 30.46 |
| Al_2_O_3_/i-Ti_3_C_2_T_x_-19.6 | Open state | 1.907 | 41.19 |
|  | Working state | 1.058 | 31.75 |
| Al_2_O_3_/i-Ti_3_C_2_T_x_-12.25 | Open state | 3.304 | 46.76 |
|  | Working state | 3.128 | 40.30 |
| i-Ti_3_C_2_T_x_ | Open state | 1.903 | 44.50 |
|  | Working state | 1.767 | 40.24 |

**Table S3.** The performance comparison of Al_2_O_3_/Ti_3_C_2_ with other reported electrodes.

| **Electrode materials** | **FAC (mg g^-1^)** | **AFAR (mg g^-1^ min^-1^)** | **Ref.** |
| --- | --- | --- | --- |
| Al_2_O_3_/i-Ti_3_C_2_T_x_ | 69.69 | 2.32 | - |
| Ni/MXene (Ti_3_AlC_2_) | 59 | 0.369 | [4] |
| Cr-MOF\|\|AC | 39.84 | 0.664 | [5] |
| CZBN/PPy\|\|MnO_2_ | 55.12 | 0.919 | [6] |
| NiCoAl-LMO\|\|rGO | 11.6 | 1.16 | [7] |
| NiAl-LMO | 49.28 | 0.548 | [8] |
| NiFeMn-LMO | 16.7 | 1.67 | [9] |
| CA | 24.44 | 0.041 | [10] |
| TWBAC | 2.83 | 0.024 | [11] |
| JFAC | 0.13 | 0.001 | [12] |
| LASAC | 2.7554 | 0.023 | [13] |
| BC | 1.28 | 0.011 | [14] |
| PANI-CNT | 9.88 | 0.329 | [15] |
| TiO_2_-loaded AC | 3 | 0.05 | [16] |
| La-AC | 5.93 | 0.039 | [17] |
| Bi/rGO | 9.12 | 0.152 | [18] |
| rGO/HA | 3.99 | 0.017 | [19] |

**Reference**

[1] S. Pervez, M. Z. Iqbal, *Small* **2023**, https://doi.org/10.1002/smll.202305059.

[2] W. Wang, P. Ma, H. Li, *Desalination* **2023**, *564*, 116798, https://doi.org/https://doi.org/10.1016/j.desal.2023.116798.

[3] a) G. Wang, T. Yan, J. Shen, J. Zhang, D. Zhang, *Environmental Science & Technology* **2021**, *55* (17), 11979, https://doi.org/10.1021/acs.est.1c03228; b) J. Wang, J. Yuan, H.-w. Gao, F. Yu, J. Ma, *Chem. Eng. J.* **2024**, *480*, 147986, https://doi.org/https://doi.org/10.1016/j.cej.2023.147986.

[4] G. Bharath, A. Hai, K. Rambabu, T. Pazhanivel, S. W. Hasan, F. Banat, *Chemosphere* **2021**, *266*, https://doi.org/10.1016/j.chemosphere.2020.129048.

[5] F. Yu, X. Zhang, P. Liu, B. Chen, J. Ma, *Small* **2022**, https://doi.org/10.1002/smll.202205619.

[6] H. Kang, D. Zhang, X. Chen, H. Zhao, D. Yang, Y. Li, M. Bao, Z. Wang, *Water Research* **2023**, *229*, https://doi.org/10.1016/j.watres.2022.119441.

[7] D. Li, S. Wang, G. Wang, C. Li, X. Che, S. Wang, Y. Zhang, J. Qiu, *Acs Applied Materials & Interfaces* **2019**, *11* (34), 31200, https://doi.org/10.1021/acsami.9b10307.

[8] Z. Bai, C. Hu, H. Liu, J. Qu, *Journal of Colloid and Interface Science* **2019**, *539*, 146, https://doi.org/10.1016/j.jcis.2018.12.062.

[9] G. Wang, D. Li, S. Wang, Z. Zhao, S. Lv, J. Qiu, *Separation and Purification Technology* **2021**, *254*, https://doi.org/10.1016/j.seppur.2020.117667.

[10] X. Zhang, Y. Li, Z. Yang, P. Yang, J. Wang, M. Shi, F. Yu, J. Ma, *Separation and Purification Technology* **2022**, *297*, https://doi.org/10.1016/j.seppur.2022.121510.

[11] M. S. Gaikwad, C. Balomajumder, *Chemosphere* **2017**, *184*, 1141, https://doi.org/10.1016/j.chemosphere.2017.06.074.

[12] J. Elisadiki, Y. A. C. Jande, T. E. Kibona, R. L. Machunda, *Ionics* **2020**, *26* (5), 2477, https://doi.org/10.1007/s11581-019-03372-z.

[13] M. S. Gaikwad, C. Balomajumder, *Separation and Purification Technology* **2018**, *195*, 305, https://doi.org/10.1016/j.seppur.2017.12.006.

[14] Q. Dong, D. Yang, L. Luo, Q. He, F. Cai, S. Cheng, Y. Chen, *Separation and Purification Technology* **2021**, *257*, https://doi.org/10.1016/j.seppur.2020.117932.

[15] L. Peng, H. Peng, C.-T. Hung, D. Guo, L. Duan, B. Ma, L. Liu, W. Li, D. Zhao, *Chem* **2021**, *7* (4), 1020, https://doi.org/10.1016/j.chempr.2021.01.001.

[16] P. Wu, L. Xia, M. Dai, L. Lin, S. Song, *Colloids and Surfaces a-Physicochemical and Engineering Aspects* **2016**, *502*, 66, https://doi.org/10.1016/j.colsurfa.2016.05.020.

[17] D. R. Martinez-Vargas, E. R. Larios-Duran, J. R. Rangel-Mendez, L. F. Chazaro-Ruiz, *Electrochimica Acta* **2022**, *425*, https://doi.org/10.1016/j.electacta.2022.140707.

[18] X. Min, M. Zhu, Y. He, Y. Wang, H. Deng, S. Wang, L. Jin, H. Wang, L. Zhang, L. Chai, *Chemosphere* **2020**, *251*, https://doi.org/10.1016/j.chemosphere.2020.126319.

[19] G. Park, S. P. Hong, C. Lee, J. Lee, J. Yoon, *Journal of Colloid and Interface Science* **2021**, *581*, 396, https://doi.org/10.1016/j.jcis.2020.07.108.
